# The Evolution of Trait Disparity during the Radiation of the Plant Genus *Macrocarpaea* (Gentianaceae) in the Tropical Andes

**DOI:** 10.3390/biology10090825

**Published:** 2021-08-25

**Authors:** Julien C. Vieu, Darina Koubínová, Jason R. Grant

**Affiliations:** Institute of Biology, University of Neuchâtel, Rue Emile Argand 11, 2000 Neuchâtel, Switzerland; julien.vieu@bluewin.ch (J.C.V.); jason.grant@unine.ch (J.R.G.)

**Keywords:** radiation, niche conservatism, adaptation, trait disparity, montane forests, Andes, *Macrocarpaea*

## Abstract

**Simple Summary:**

The evolutionary radiation of lineages can be caused by several mechanisms. We investigated the evolutionary history of the plant genus *Macrocarpaea* (Gentianaceae) from the middle elevation montane forests of the Tropical Andes, one of the most species-rich areas of the world. We tested several evolutionary hypotheses based on molecular, morphological and climatic data, using phylogenetic comparative methods. In this paper, we identify the processes dominating the diversification history of *Macrocarpaea*, especially the *M. micrantha* clade.

**Abstract:**

The evolutionary processes responsible for the extraordinary diversity in the middle elevation montane forests of the Tropical Andes (MMF; 1000–3500 m) remain poorly understood. It is not clear whether adaptive divergence, niche conservatism or geographical processes were the main contributors to the radiation of the respective lineages occurring there. We investigated the evolutionary history of plant lineages in the MMF. We used the vascular plant genus *Macrocarpaea* (Gentianaceae) as a model, as it consists of 118 morphologically diverse species, a majority of which are endemic to the MMF. We used a time-calibrated molecular phylogeny and morphological and climatic data to compare a set of evolutionary scenarios of various levels of complexity in a phylogenetic comparative framework. In this paper, we show that the hypothesis of adaptive radiation for *Macrocarpaea* in the MMF is unlikely. The genus remained confined to the upper montane forests (UMF > 1800 m) during more than a half of its evolutionary history, possibly due to evolutionary constraints. Later, coinciding with the beginning of the Pleistocene (around 2.58 Ma), a phylogenetically derived (recently branching) clade, here referred to as the *M. micrantha* clade (25 species), successfully colonized and radiated in the lower montane forests (LMF < 1800 m). This colonization was accompanied by the evolution of a new leaf phenotype that is unique to the species of the *M. micrantha* clade that likely represents an adaptation to life in this new environment (adaptive zone). Therefore, our results suggest that niche conservatism and geographical processes have dominated most of the diversification history of *Macrocarpaea*, but that a rare adaptive divergence event allowed a transition into a new adaptive zone and enabled progressive radiation in this zone through geographical processes.

## 1. Introduction

Understanding the evolutionary mechanisms responsible for the fact that some lineages are more species-rich than others is a fundamental aim of the research in evolutionary biology. The ecological theory of adaptive radiation estimates that ecological opportunity is a primary factor regulating the tempo of lineage diversification [1]. Ecological opportunity can be perceived as the availability of under-exploited resources that can arise from the extinction of competitors, dispersal into new habitats or acquisition of a key innovation that allows organisms to explore new ecological niches [2,3]. During the phase of niche exploration, divergent selection is thought to promote bursts of species divergence (diversification), together with a rapid accumulation of phenotypic diversity (disparification [4]). As niche space becomes progressively saturated, rates of diversification and disparification are expected to slow down [5]. Classical examples of adaptive radiations often involve organisms located on islands or island-like habitats (i.e., lake and mountaintops), such as Galapagos Darwin’s finches in the Galapagos Islands [6]; the Caribbean *Anolis* Daudin, 1802 lizards [7]; or the African rift lake cichlid fishes [8]; or even plants such as the Hawaiian silverswords [9]. However, more recently, cases of adaptive radiations on continents, e.g., in the vertebrates in North America, Neotropics or in the oceans, have also been documented [10,11,12].

Adaptive radiations have been proposed to be responsible for much of the biodiversity on Earth [13,14]. However, the ubiquity of the link between diversification and disparification rates predicted by the adaptive radiation theory has been recently challenged [15,16]. Several studies have revealed that bursts of diversification are not necessarily associated with bursts of morphological disparity [17,18,19,20]. This suggests that adaptive divergence is not the only evolutionary process that can lead to episodes of rapid diversification in a clade. Indeed, geographical processes arising from the interactions between habitat spatial fragmentation, lineage ecological niche stasis (niche conservatism) and low dispersal ability also have the potential to produce bursts of diversification by inducing repeated allopatric speciation [21,22]. Under this type of non-adaptive radiation, diversification and disparification rates are expected to be decoupled from one another, such that rapid species diversification is not accompanied by rapid trait disparification [23]. As noted later [22], natural selection is not absent from this process. Rather, stabilizing selection constrains the evolution of the ecological niche over time; thus, it contributes to preventing secondary contact between allopatric populations separated by unfavorable habitats that can ultimately lead to speciation. To date, non-adaptive radiations have perhaps received less attention than adaptive ones but compelling examples have been documented in several animal species, such as North American *Plethodon* Tschudi, 1838 salamanders [24]; damselflies [25], African *Nothobranchius* Peters, 1868 killifish [26]; or plants Aegean *Nigella* L., 1753 [27] (but see [21]). Despite recent advances, the relative contribution of adaptive (ecological based divergent selection) and non-adaptive processes (stabilizing selection and drift) to shaping biodiversity are poorly understood.

The middle-elevation mountain forests of the Tropical Andes, spreading from Northern Venezuela to Northern Bolivia (MMF; 1000–3500 m, lower LMF < 1800 m and upper UMF 1800–3200 m montane forests and subpáramo shrub forests >3200 m; moist with >1500 mm per year), offer an excellent study system to address these questions. First, they harbor a tremendous amount of biodiversity, including many diverse lineages rich in endemics [28], suggesting that radiations have been common in the MMF [29]. Secondly, the complex physiography of the Andes with deep valleys and high peaks represents a large area with steep and extended ecological gradients associated with elevation as well as physical barriers to gene flow. Ecological gradients are thought to promote adaptive divergence [30], whereas physical barriers to gene flow can promote geographic isolation and may lead to allopatric speciation [31]. Third, the MMF evolved relatively recently, likely during the late Miocene as the Andes reached sufficient elevation to trigger high year-round precipitation on the eastern slopes [32,33,34]. These vast and recently emerged habitats might have represented considerable new ecological opportunities for the regional biodiversity pool to colonize and to adapt to. Finally, the recent history of the MMF has been marked by the interglacial–glacial periods during the Pleistocene climatic oscillations (PCO, 2.58–0.01 Ma). They caused dramatic fluctuations of the altitudinal range and cycles of contraction and re-expansion of the main vegetation belts during the glacial and interglacial periods, respectively [35,36], as well as associated population fragmentation (glacial periods) and re-expansion (interglacials; [37]). Thus, the PCO potentially induced ideal conditions to promote extensive allopatric speciation at the scale of the whole MMF territory.

Several studies focusing on a variety of animal groups have used the MMF system to study the evolutionary processes responsible for species divergence and evolutionary diversification. Whereas examples of adaptive divergence have been documented (butterflies [38]), most studies point to a preponderance of niche conservatism and non-adaptive geographical processes (allopatric speciation) as the main driver of species divergence in the MMF (birds [39,40], butterflies [41], frogs [42] and mice [43]). By comparison, the processes and the mechanisms involved in plant diversification in the MMF have been much less studied. Recently, several studies have investigated plant diversification dynamics in the MMF [29,44,45,46,47]. They have suggested that colonization and/or range expansion across emerging MMF could have represented a key opportunity for these plant groups to diversify through rapid allopatric speciation. This would signify that non-adaptive processes were privileged over the adaptive ones. There is compelling evidence for accelerated species diversification rates in these clades. However, the majority of the mentioned studies has not estimated rates of ecological and/or morphological disparification through time to investigate a potential concomitant role of adaptive divergence during the diversification of these clades (but see [46]).

**Figure 1:**
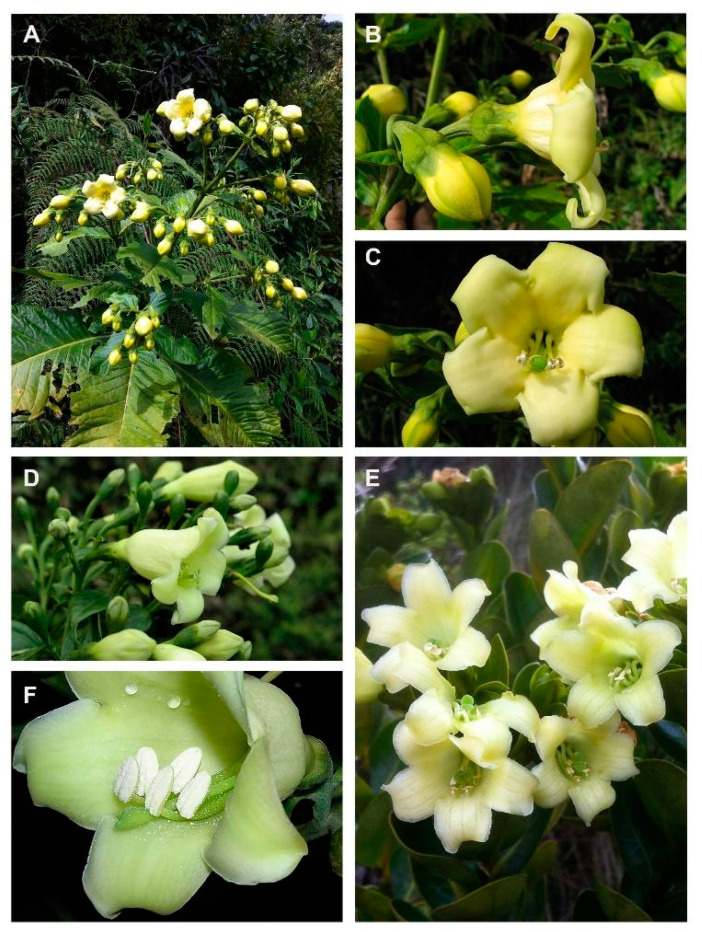
(**A**–**C**) *Macrocarpaea jactans* J. R. Grant, (**D**) *M. noctiluca* J. R. Grant and Struwe, (**E**) *M. subsessilis* Weaver and J. R. Grant, (**F**) *M. apparata* J. R. Grant and Struwe. Copyright for all photos: Jason R. Grant.

**Figure 2:**
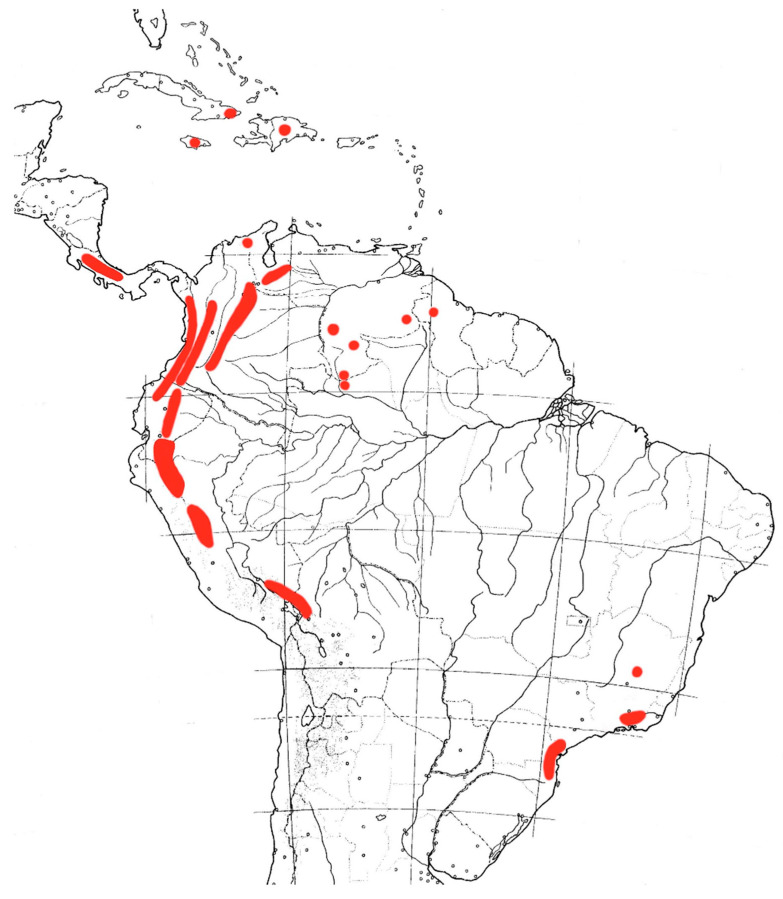
The map shows the known distribution of the plant genus *Macrocarpaea* in the Neotropics—in red.

In this study, we explored the potential contributions of adaptive divergence and niche conservatism to plant diversification in the MMF, using the genus *Macrocarpaea* (Griseb.) Gilg (Gentianaceae, Figure 1). The genus comprises 118 species [48,49,50,51,52,53,54,55,56,57,58,59,60,61,62] and two hybrids, and there are still more new species under investigation, mostly from Peru. They are exclusively found in mountainous regions of the Neotropics (especially in the Andes, the Guayana Highlands, Mesoamerica, the Greater Antilles and the montane Atlantic forests of Brazil; Figure 2 [48]). Ninety-seven species (82% of the total number) are endemic to the Andean MMF. A recent study showed that the clade, including all *Macrocarpaea* species from the MMF, was diversified by following the classical diversification trajectory expected under radiation (early burst followed by a slowdown; [29]). The authors suggested that rapid allopatric speciation during rapid range expansion of the genus across the MMF facilitated the radiation. *Macrocarpaea* displays substantial morphological variation, with species ranging from small herbs/shrubs with small coriaceous leaves and <1 m tall to 10 m tall trees with large leaves. Although the genus spans a broad elevational range (500–3500 m), most species are restricted to about 500 m elevation bands. If these morphological and ecological trait differences evolved synchronously with the burst of species diversification, it would indicate that the radiation of *Macrocarpaea* in the MMF has an important adaptive component. 

Alternatively, it is possible that the trait differences accumulated later. For example, niche evolution could have been constrained or slow in the early history of the MMF clade, but during the PCO with repeatedly changing climates, species might have adapted to distinct elevation bands instead of migrating. This would be associated with enhancing trait evolution toward the tip of the tree. One of the possibilities might also be evolution driven by pollinators [46]. *Macrocarpaea* is a generalist in terms of pollinators as it has both nocturnal (bats and insects) and diurnal pollinators (hummingbirds and insects).

Notably, a large majority of *Macrocarpaea* MMF species occur in the upper montane forest (UMF > 1800 m [63]). However, in a phylogenetically derived (recently branching) clade recovered in a previous study [29], the majority of the species (19 out of 25) occurred in the lower montane forest (LMF < 1800 m). Here, we refer to this lineage as the *M. micrantha* Gilg clade, because of the early branching lineage/species of this clade. This occurrence in the LMF relatively depauperate from others *Macrocarpaea* lineages could indicate that the ancestor of the *M. micrantha* clade invaded the LMF as a new adaptive zone *sensu* Simpson [64]. In consequence, the trait evolutions in the *M. micrantha* clade might have been decoupled from the rest of the MMF clade. Alternatively, trajectories of trait evolution could have been elevation belt-specific and all the lineages occurring in the LMF including the few species outside of the *M. micrantha* clade might have followed the same evolutionary trajectories. Finally, it is also possible that the pace and trajectories of trait evolution have been influenced by region-specific conditions. The Amotape-Huancabamba zone (AHZ), a region located in Southern Ecuador and Northern Peru (between 3° and 8° S), is the center of diversity for many Andean plant lineages in the MMF, including *Macrocarpaea* [65,66]. It has been proposed that the exceptional habitat heterogeneity of the AHZ enhanced adaptive divergence more than in any other region of the MMF [65,66]. According to this hypothesis, *Macrocarpaea* lineages from the AHZ should display faster trait evolution than lineages from elsewhere.

Here, we use a previously generated [29] time-calibrated phylogeny for the genus *Macrocarpaea* based on six molecular markers and compile morphological and climatic datasets in a phylogenetic comparative method (PCM) framework to (1) assess the contribution of adaptive divergence during the radiation of *Macrocarpaea* in the MMF (if adaptive divergence fueled the radiation, we expect to detect a burst of disparification in the early history of the MMF clade); (2) test whether traits evolution for *Macrocarpaea* in the MMF followed a constrained or an unconstrained mode of evolution; and (3) evaluate whether the constraint or the rate of evolution was constant in time and uniform across all species, or varied according to (i) climate dynamism history, (ii) regional or (iii) elevation band-specific condition, or (iv) clade-specific history.

## 2. Materials and Methods

### 2.1. Time-Calibrated Phylogeny

We used a previously generated time-calibrated phylogeny of *Macrocarpaea* based on six molecular markers (ITS, 5S-NTS, *rpl16*, *Trnl*, *trnH-psbA* and *rpl32*) that included 75 of the 118 currently recognized species in the genus [29]. The samples originated from field sampling or herbarium specimens. The maximum clade credibility tree (MCC tree) and a random sample of 100 trees from the posterior distribution of the Bayesian analyses were pruned to remove all non-Andean species. After pruning, the phylogeny included 63 Andean species representing 65% of the MMF diversity of the genus (63/97).

### 2.2. Morphological Data

Morphological data for 55 Andean *Macrocarpaea* species were obtained from measurements of herbarium specimens that were performed as a part of the species descriptive taxonomic research based on all the available herbarium specimens (2154 sheets). The number of specimens examined varied greatly between species (min = 2, max = 215, median = 28), resulting from a combination of the variation in sampling effort and the abundance of each species. Six traits were selected by using the following criteria: (a) reflecting the morphological variation displayed by the genus, (b) having data measured for a high proportion of species included in the phylogeny and (c) and having relatively straightforward plant functional interpretations. (1) Plant height, (2) leaf length and (3) leaf width describe the vegetative components of the plants and their variation and likely reflect evolution driven by adaptation to the environment. (4) Calyx width, (5) corolla width at mouth and (6) corolla length describe reproductive biological components and their variation potentially as a result of evolution driven by pollinators. The measurements used are estimates of ranges of size (minimum and maximum) for the diverse traits. From these, we estimated the mean by assuming that it was equal to the center of the range. We acknowledge a potential source of inaccuracy around this assumption. The overall proportion of missing data in the dataset is 6%. Out of the 55 species examined, for 9 species, data for two traits were missing (*M. angelliae* J. R. Grant and Struwe; *M. arborescens* Gilg and *M. catherineae*, J. R. Grant; *M. chthonotropa* J. R. Grant; *M. gondoloides* J. R. Grant; *M. luteynii* J. R. Grant and Struwe; *M. micrantha* and *M. pacifica* J. R. Grant; and *M. quechua* J. R. Grant). These traits were corolla width at the mouth (9 species), corolla length (5 species) and calyx width (4 species). To avoid removal of species with missing data, we imputed missing data by using a method that considers the phylogenetic relationship to improve the imputation process [67]. Data were log-transformed prior to the imputation to reduce scale variations of the distinct variables. We used the PVRdecomp function from the R software package PVR v. 0.2.1 [68] in R v.3.2.0 [69] to decompose the phylogenetic distance matrices into a set of orthogonal eigenvectors. We kept the first ten eigenvectors (representing 64% of the variation in the phylogenetic distances among species) and appended them to the trait matrix that already contained 12 columns (minimum and maximum values for 6 traits). Imputation was done on this hybrid matrix in the R package missForest v.1.4 [70] using the options maxiter = 20 and ntree = 1000. MissForest stopped after 6 iterations and returned a relatively low error estimate (normalized root squared error, NRMSE = 0.042). After the imputation process, species means were computed from the log-scale minimum and maximum values for each trait. Phylogenetic correlations between plant size and the other morphological traits were tested by applying a correlation test on the phylogenetic independent contrasts (PIC). Among the five traits, four displayed a significant positive correlation with plant size (r = 0.31–0.45, *p* = 0.0004–0.019), whereas the correlation with calyx width did not show such significant correlation (r = 0.23, *p* = 0.094). The five shape trait measurements were size-corrected using phylogenetic regression on plant size that account for the phylogenetic non-independence of species, within the R package phytools v. 0.4.56 [71]. To do this, the phylogeny was pruned to include only the species with morphological data. Residuals from the phylogenetic regression were extracted to conduct a phylogenetic principal-components analysis (pPCA) that also accounted for the phylogenetic non-independence, on the covariance matrix [72]. We note that the preparatory steps described above were repeated for each tree (n = 100) in the posterior sample. All subsequent analyses relative to morphological evolution were conducted on the following: (1) log plant size (in log-mm), (2) the morphology pPCA1 (leaf size) and (3) the morphology pPCA2 (flower size).

### 2.3. Ecological Data

Occurrence data were compiled from geo-referenced herbarium specimens. The occurrences were used to extract elevation and climatic data from the WorldClim dataset v. 1.0 [73] that comprises 19 bioclimatic (BioClim) variables. They describe average and seasonal variation in temperature and precipitation at a 30-s resolution. The extraction process was performed by using the R package RASTER v. 2.3.40 [74]. Species means were computed for latitude, longitude, elevation and the 19 BioClim variables. This dataset of 22 variables and the phylogeny pruned to include species with climatic data only (62 species) were used to conduct a pPCA on the correlation matrix because sizes and units of the variables included were distinct. Again, all the preparatory steps were repeated for each tree (n = 100) in the posterior sample. Subsequent analyses related to the climatic niche evolution of *Macrocarpaea* species were conducted on (1) the climatic pPCA1 (altitudinal niche) and (2) the climatic pPCA2 (latitudinal niche).

### 2.4. Functional Data

Among plant functional traits, specific leaf area (SLA, defined as fresh leaf area/dry mass), which is associated with carbon capture, is commonly used as a proxy for contrasting plant physiological strategies [75]. SLA was measured for 31 species from specimens freshly collected in the field. A total of 774 individuals were measured, with an average of 20 individuals per species (range: 3–87). Mean SLA was computed for each species from the log-transformed individual data (log mm^2^·mg^−1^) and was used in subsequent analysis.

### 2.5. Disparification, Evolutionary Models and Phylogenetic Signal

To investigate the evolution of trait and ecological disparification through time we computed the morphological disparity index (MDI [76]) for the six traits described above on the MCC tree with the R package GEIGER v. 2.0.5 [77]. The statistical significance of deviations from the BM expectation was assessed by using 2000 simulations for each trait. 

To compare various scenarios for the evolution of traits and climatic niches of *Macrocarpaea* in the Andes, we considered a set of 11 models with varying levels of complexity:

(1) A simple Brownian model (BM) that describes trait evolution driven by neutral processes such as genetic drift [78]. We expected this model to perform better if trait evolution was unconstrained and did not show any specific pattern related to time, elevation belts, taxonomy or geography;

(2) An Ornstein–Uhlenbeck model with a single optimum (OU) that describes trait evolution driven by stabilizing selection toward a single adaptive zone [78,79]. A preference for this model would indicate that evolutionary constraints homogeneous across the whole phylogeny have governed traits evolution;

(3) An early burst model (EB) that corresponds to a niche-filling process during adaptive radiation [16]. If the radiation of *Macrocarpaea* in the Andes was an adaptive one, we expect this model to perform better;

(4) A two-rate BM model (SHIFT) with the rate of evolution allowed to change after a fixed point in time set to be 2.6 Ma [80]. The preference of this model would indicate that the Pleistocene climatic oscillations (PCO) have induced a slow-down or an increase in the rate of evolution;

(5) An ecological release model (ER) that assumes a constrained evolution under an OU model prior to a fixed point in time, followed by un unconstrained evolution under a BM model [81]. The selection of this model would suggest that Pleistocene climatic oscillations have released species from their ancestral adaptive optima and allowed them to explore wider morphospace and ecospace;

(6) A two-rate BM model (BMM Regime) where species from the lower montane forest (LMF, <1800 m) and the upper montane forest (UMF, >1800 m) can have different rates of trait evolution. A better fit of this model would indicate that species from the two main vegetation belts of MMF have distinct rates of trait evolution [63];

(7) An OU model with two optima (OUM Regime) where species from the LMF and UMF can have distinct adaptive optima. The selection of this model would indicate that species from the two vegetation belts have evolved under divergent selection (convergence);

(8) A two-rate BM model (BMM Clade) where species from the *M. micrantha* clade can have a different rate of evolution from the rest of the phylogeny. The choice of this model would indicate a change in the tempo of trait evolution for the species of the *M. micrantha* clade;

(9) An OU model with two optima (OUM Clade) where species from the *M. micrantha* clade can have a unique adaptive optimum from the rest of the phylogeny. A better fit of this model would indicate that the species of the *M. micrantha* clade have shifted toward a new adaptive optimum; 

(10) A three rate BM model (BMM Geo) where species from the Northern Andes, the Amotape-Huancabamba zone and the Central Andes may have contrasting rates of evolution. The choice of this model would suggest that the rate of trait evolution is region-specific; 

(11) Finally, an OU model with three optima (OUM Geo) where species from the three regions of the MMF can have divergent adaptive optima. The selection of this last model would indicate that the direction of stabilizing selection is region-specific. 

We note that the BM or OU are a particular case of the complex model described above where rates or optima for the different part of the tree are equal. The BM model is also a particular case of the OU model when the selection parameter (α) tends toward zero.

Inference of the habitat (LMF vs. UMF) and the geographical area (the Northern Andes, Amotape-Huancabamba zone and Central Andes) across the phylogenies was performed by using a marginal ancestral state reconstruction method in the R package phytools v. 0.4.56 with a symmetric transition matrix [71]. Time slices, regimes, clade and geography were mapped onto the trees, using the “paintBranches” function from phytools. The models were fitted for each trait/climate niche in the R package MvMORPH 1.0.4 [82] that takes as input trees with mapped characters. All the analyses and preparation steps were performed on the MCC tree and repeated on the posterior sample of 100 trees to account for phylogenetic uncertainty. Model performances were compared by using corrected Akaike information criterion (AICc) scores.

To evaluate the statistical power of our phylogeny to discriminate among the models, we applied the Monte Carlo–based approach [83]. The method first estimated parameters for both models to be compared with the real data. Then two datasets were generated by simulating a large number of trait samples under each model and using the parameters estimated by using the real data. For each sample in each dataset, the parameters were re-estimated by using the two models, allowing us to compute a likelihood ratio, δ (see Equation (3) [83]). Finally, the two distributions of δ generated were compared between them and with the likelihood ratio observed on the real data (δ_0_). The probability of rejecting the “simpler” model (or the poorer fit based on AICc) was obtained by computing the proportion of the distribution of δ_s1_ (computed on the dataset simulated under model 1, the poorer fit model) that was greater than δ_0_. The “power” of the test was evaluated by computing the proportion δ_s2_ (computed on the dataset simulated under the model 2, the fittest model) that was greater than the 0.95 quantiles of the distribution of δ_s1_. For each trait, we applied this approach to verify the ability of our data to discriminate between the two best-fitting models according to the AICc. For each model considered, 2000 samples of traits were simulated on the MCC tree with the function “OUwie.sim” in the R package OUwie 1.45 [84].

Finally, we extracted the selective parameter (α) estimated under the OU model to compute the phylogenetic half-life (t_1/2_ = ln2/α) for each trait. The half-life is expressed in the same time units as the time-calibrated trees used to compute it (here in Myr) and represents the amount of time necessary for a clade to move halfway out from the adaptive optimum of its ancestor [79]. In addition, we reported the stationary variance (*V_y_* = σ/2*α) estimated under the OU model (and OUM when preferred) that quantified the relative strength of drift around the adaptive optimum (σ) vs. directional selection toward the optimum (α).

## 3. Results

In the pPCA conducted with the morphological traits’ dataset, we retained the two first axes that collectively explained 88% of the variation and have relatively straightforward biological meaning. Leaf traits had high negative loadings on the first pPCA axis that most likely described variations in leaf size. In contrast, floral traits had relatively high negative loadings on the second pPCA axis that likely described variations in a floral shape. Trait loadings and the proportion of variance explained by the two first pPCA axes for the analysis based on the MCC tree are shown in Table 1.

After excluding duplicated localities, the occurrence dataset contained a total of 464 occurrences for 62 species. Overall, the number of occurrences varied greatly between species (min = 1, max = 36 and median = 5).

The dataset of 22 ecological variables and the phylogeny pruned to include species with climatic data only (62 species, Supplementary Materials Figure S1) were used to conduct a pPCA on the correlation matrix because sizes and units of the variables included were distinct. The two first axes of the pPCA explained 68% of the variation and were retained. Table 2 reports the traits loadings and the proportion of variance explained by the two first pPCA axes for the analysis based on the MCC tree. The variables with strong loadings on the first axis were altitude and BioClim variables, representing average, minimum and maximum of temperatures and precipitation. The variables with strong loadings on the second axis were latitude, longitude and the BioClim variables, representing seasonal variation in temperatures. Thus, the pPCA axis one represented the climatic variation related to the elevation range of the species and most likely described the altitudinal niche of species that potentially constraint their migration toward adjacent elevation zones. On the other hand, the pPCA axis two represents climatic variations related to the latitudinal position (as the Andes have a banana shape, absolute latitude and absolute longitude were strongly negatively correlated, r = −81) and likely described the regional environmental niche of species that potentially constrained their migration toward adjacent regions. We acknowledged that climatic variables represented extrinsic factors that were not directly inherited by descent [85]; nevertheless, they indirectly reflected intrinsic physiological tolerances of species that are of evolutionary relevance.

**Figure 3:**
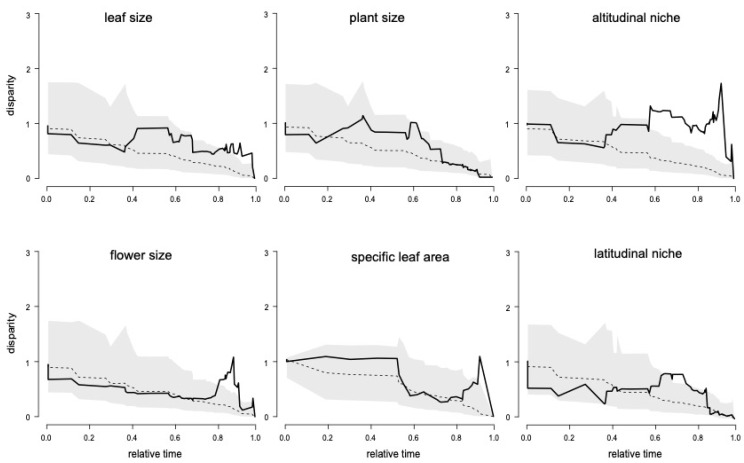
Mean subclade disparity through time (DTT) for the 6 traits investigated in this study (solid line), based on the maximum clade credibility (MCC) tree of the Andean species of *Macrocarpaea*. The dashed lines indicate the median subclade DTT based on 2000 simulations of character evolution under a Brownian motion of evolution. The gray shaded areas indicate the 95% DTT range for the simulated data. The x-axis is the relative time, with x = 0 being the root of the tree and x = 1 the tips.

Plots of the observed and simulated disparities against relative time from the root of the tree for the Climate pPCA1 and pPCA2 are displayed in Figure 3. They indicated that, in the initial history of the Andean *Macrocarpaea* clade, disparification was not different from the expectation under a BM model of evolution for all the traits investigated. Later (~3.2 Ma), the disparity became much greater than the BM expectation for the altitudinal niche. The disparity also became moderately greater toward more recent times for leaf size, flower size and SLA. Disparity below the median of the BM expectation was observed only in the initial history for the latitudinal niche, but it remained within the 95% interval for the BM expectation. This was also reflected by the MDI statistic (Supplementary Materials Figure S3) that was moderately negative only for the latitudinal niche, but it was not statistically different from the BM expectation (MDI = −0.011, *p* = 0.74). The MDI statistic compared the observed trait disparity through time (DTT) to the median of a distribution of DTT simulated under a Brownian motion model of evolution (BM) on the phylogeny. Negative values of MDI indicate that disparity is principally distributed among subclades and is considered to be the hallmark of adaptive radiation [76]. On the other hand, positive values of MDI indicate that disparity is distributed within subclades. The evolutionary interpretation of this second pattern is less clear but would tend to indicate that each subclade explored a higher proportion of the niche/trait space than expected from a BM model. Negative MDI was the expected pattern under adaptive radiation [76]. Here, none of the variables investigated matches this prediction. Positive and statistically distinct MDIs from the BM expectation were recovered for the altitudinal niche (MDI = 0.399, *p* = 0.98). This indicates that species within the various subclades occupy a larger proportion of the altitudinal niche than expected from a BM evolution. The MDI for the other variables was moderately positive to positive (MDI = 0.007–0.185), but none was significantly different from the BM expectation (*p* = 0.74–0.91).

**Figure 4:**
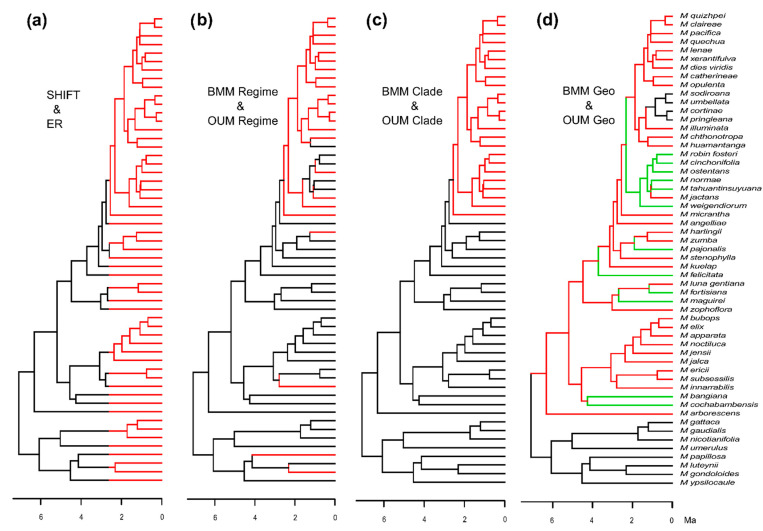
Mapping of time slices (**a**), regimes (**b**), taxonomy (**c**) and geography (**d**) on the MCC tree of the Andean species of *Macrocarpaea* that have morphological data (55 species) that were used to build the distinct models of trait evolution. (**a**) The mapping depicts part of the tree older than the Pleistocene climatic oscillations (PCO, >2.6 Ma) in black from those contemporaneous to the PCO in red. The SHIFT is a two rate Brownian model (BM) of trait evolution were black portions of the tree share the same rate of evolution σ_1_ and red portions have a different rate σ_2_. The ER (for ecological release) is an Ornstein–Uhlenbeck model (OU) applied to the black part of the tree, followed by a BM applied to the red branches. (**b**) Black branches correspond to species occurring in the upper montane forest (UMF, >1800 m) and red branches to species occurring in the lower montane forest (LMF, <1800 m). States for internal branches were estimated by using maximum likelihood methods (ML, see Methods for details). The BMM Regime is a two-rate regime specific BM model, were black branches have a rate σ_1_ and red branches have another rate σ_2_. The OUM Regime is a two optimum regime specific OU model, where black branches have an optimum θ_1_ and red branches have another optimum θ_2_. (**c**). Red branches correspond to the clade of the *M. micrantha* clade and black branches correspond to parts of the tree outside the *M. micrantha* clade. The BMM Clade is a two-rate clade-specific BM model (black = σ_1_, red = σ_2_) and the OUM Clade is a two-optima clade-specific OU model (black = θ_1_, red = θ_2_). (**d**) Black, red and green branches correspond to species occurring in the Northern Andes, the Amotape-Huancabamba zone and the Central Andes, respectively. States for internal branches were inferred by using ML methods. The BMM Geo is a three rates region specific BM model (black = σ_1_, red = σ_2_, green = σ_3_) and the OUM Geo is a three optima region specific OU model (black= θ_1_, red = θ_2_, green = θ_3_).

Figure 4 illustrates the mapping of traits onto the MCC tree, including all the species for which we obtained morphological data (compare with Supplementary Materials Figure S1—climatic data and Supplementary Materials Figure S2—SLA data). The maximum likelihood ancestral state estimation for the regime consistently reconstructed the MRCA of Andean *Macrocarpaea* as occurring in the UMF and the most recent common ancestor (MRCA) of the *M. micrantha* clade as occurring in the LMF. Concerning the geography, the Andean MRCA was ambiguously reconstructed as occurring either in the Northern Andes or the AHZ in agreement with previous work [29,61].

**Figure 5:**
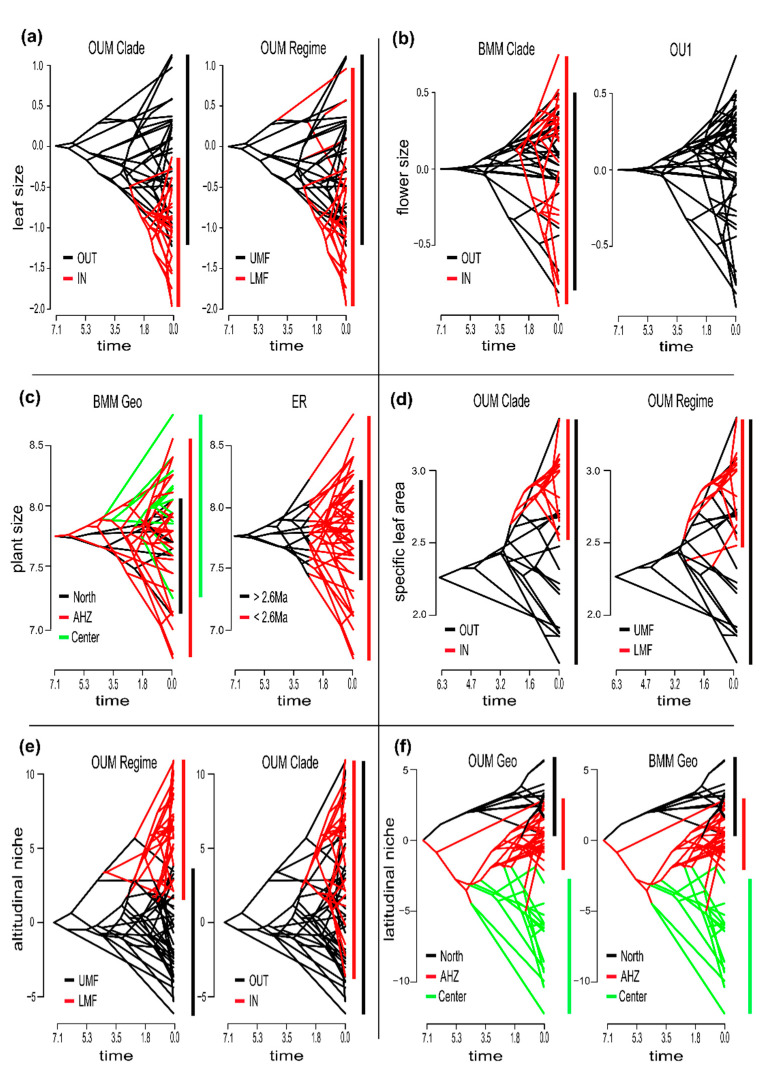
Phenograms reconstructed by using the R package phytools (Revell, 2012) for the 6 traits investigated in the study. The x-axes represent the time in Myr and the y-axes the trait values. The method uses a maximum likelihood approach to infer the ancestral value at internal nodes. For each trait, two phenograms are displayed with branch colors representing alternative mappings that were used to fit the two best-fitting models (see Table 3 for models scores). Vertical lines on the right of the phenograms represent the trait range value for each mapped state. (**a**) For leaf size the best fitting model is the OUM Clade (see caption of the Figure 4 and Methods for details concerning the models) followed by the OU1, but here we decided to represent the OUM Regime instead (3^rd^ best model), for discussion purpose. IN and OUT mean inside and outside the *M. micrantha* clade respectively. UMF and LMF refer to the upper (>1800 m) and lower (<1800 m) montane forest respectively. (**b**) For flower size the two best models are the BMM Clade and OU1. (**c**) For plant size, the BMM Geo and the ER are the two best models. North, AHZ, and center refer to the Northern Andes, the Amotape-Huancabamba zone and the Central Andes respectively whereas >2.6 Ma and <2.6 Ma refer to the part of the trees older and contemporaneous with the Pleistocene climatic oscillations respectively. (**d**) For specific leaf area the OUM Regime and OUM Clade obtain almost equivalent fit. (**e**) For the altitudinal niche the OUM Regime obtained the best fit and is followed by the OUM Clade. (**f**) For the latitudinal niche, the best-fitting model is the OUM Geo, followed by the BMM Geo.

Model comparison for the evolution of the leaf size preferred the OUM Clade model that accounted for distinct adaptive optima for the *M. micrantha* clade and the rest of the phylogeny (Table 3). The second-best model was the OU1, which had a single optimum for the whole phylogeny, but the AICc difference between these two models was large (MCC tree, ΔAICc = 7.66; median of the posterior sample, ΔAICc = 7.68), indicating strong support for the OUM Clade model over the alternative models [86]. The optimum for the *M. micrantha* clade (θ_2_; full parameter estimates are available in the Supplementary Materials Table S1) was toward larger leaves than for the rest of the phylogeny (θ_1_; see Figure 5a). The simulation procedure to estimate the power of our data to discriminate between the two best models based on the MCC tree indicated that we could reject the OU1 in favor of the OUM Clade model with high probability (*p* = 0.005; see Figure 5 for plots of the distributions of simulations) and strong power (98.9%).

Considering flower size, the best fit was to the BMM Clade model that accounted for distinct rates of evolution for the *M. micrantha* clade and the rest of the phylogeny. The second-best model was the OU1, but again, the AICc difference was large (MCC tree, ΔAICc = 7.03; median of the posterior sample, ΔAICc = 5.31). The estimated rate of flower size evolution for the *M. micrantha* clade (σ_2_) was more than four times higher than the rate for the rest of the phylogeny (σ_1_, Figure 5b). The power test indicated that we could reject the OU1 in favor of the BMM Clade with a high probability (*p* = 0.003) and strong power (94.2%).

For plant size, the BMM Geo model that allowed a region-specific rate of evolution (three rates) obtained the lowest AICc and was followed by the ER that accounted for a release of stabilizing selection during the last 2.6 Myr (MCC tree, ΔAICc = 1.84; median of the posterior sample, ΔAICc = 2.59). Simulations rejected the ER in favor of the BMM Geo (*p* = 0.024) with substantial power (88.6%). Under the BMM Geo model, the rate of plant size evolution estimated in the Amotape-Huancabamba region was almost the double (σ_2_) that of the rate in the Central Andes (σ_3_) and more than five times larger than in the Northern Andes (σ_1_, Figure 5c).

For the evolution of the SLA, the OUM regime that accounted for distinct adaptive optima for the LMF and the UMF, and the OUM Clade model obtained almost equivalent support (MCC tree, ΔAICc = 1.74; median of the posterior sample, ΔAICc = 0.091). In comparison, all the other models were outperformed (MCC tree, ΔAICc > 8; median of the posterior sample, ΔAICc > 7). Simulations based on the MCC tree shown that we cannot reject the OUM Regime in favor of the OUM Clade at the 5% level (*p* = 0.063) with a power of 88.4%. The estimates of the optimum for the UMF (θ_1_) under the OUM Regime and the optimum for the species outside of the *M. micrantha* clade (θ_1_) under the OUM Clade are close and correspond to the SLA of 9.90 and 10.00 mm^2^·mg^−1^, respectively (once transformed back to their original scales). The optimum estimated for the LMF (θ_2_) was a bit larger than the optimum for the *M. micrantha* clade (θ_2_) with the SLA of 21.20 mm^2^·mg^−1^ and 18.65 mm^2^·mg^−1^ respectively but imply in both cases selection toward leaves that are twice as thin.

The best model for the altitudinal niche was, as expected, the OUM Regime that accounted for distinct optima between the LMF and the UMF. All the other models have a much poorer fit (MCC tree, ΔAICc > 50; median of the posterior sample, ΔAICc > 50). The second-best model was the OUM Clade model that also has a large AICc difference with the next best-fitting model (MCC tree, ΔAICc > 9; median of the posterior sample, ΔAICc > 8). Not surprisingly, the OUM Clade model was rejected in favor of the OUM Regime with high probability (*p* = 0.000) and maximal power (100%).

For the latitudinal niche, the OUM Geo model that accounted for region-specific adaptive optima (three θ) was returned, as expected, as the best model (MCC tree, ΔAICc > 33; median of the posterior sample, ΔAICc > 42). The second-best model was the BMM Geo model, but in comparison, the remaining models have a much poorer fit (MCC tree, ΔAICc > 42; median of the posterior sample, ΔAICc > 36). The BMM Geo model was rejected in favor of the OUM Geo model with high probability (*p* = 0.00) and strong power (*p* = 100%). However, the observed likelihood ratio using the real data (δ_0_) was much smaller than the distribution of ratio obtained on the dataset simulated with the OUM Geo model. This indicates that the OUM Geo model might not describe properly the data. Under the BMM Geo model the rate of evolution in the Central Andes (σ_3_) was 12 times higher than in the Northern Andes (σ_1_) and more than 30 times higher than in the Amotape-Huancabamba region (σ_2_).

Overall, the EB model obtained extremely low support for all the variables investigated, and its rate change parameter (r) was always returned as null. Models that accounted for potential influences of the Pleistocene climatic oscillations (SHIFT and ER) obtained some support but only for the evolution of plant size.

The phylogenetic half-life estimated under the OU1 model for the varying traits (see Table 4) was, in general, much smaller than the age of the most recent common ancestor (MRCA) of *Macrocarpaea* in the Andes (7.16 Myr). The half-life can be used as a proxy to estimate the phylogenetic signal in a trait [87,88]. A half-life greater than the age of the MRCA of the clade under consideration indicates that the evolution of the trait is almost indistinguishable from evolution under a BM model, and therefore the phylogenetic signal is strong [89]. In contrast, when the half-life is much smaller than the age of the youngest node in the clade, the evolution of the trait is not phylogenetically correlated, and the phylogenetic signal is null. If the best model chosen for a trait during the model comparison process was an OUM model (multiple optima), we also report the estimated half-life under this model. This allows us to compare how much phylogenetic signal remains after we account for divergent adaptive regimes. This suggests a relatively low phylogenetic signal. The only exception was the regional niche that has a half-life comparable with the age of the MRCA of the clade (t_1/2_ = 5.44 Myr). This was consistent with the model comparison that preferred the BM1 over the OU1 for this trait. Interestingly, the leaf size, flower size and SLA had similar half-lives (t_1/2_ = 1.635–1.978 Myr), whereas the half-life of plant size was substantially greater (t_1/2_ = 2.925 Myr). Under the OUM Clade model (best fit), the half-life for leaf size (t_1/2_ = 0.266 Myr) collapsed to a value smaller than the youngest node in the phylogeny (age = 0.360 Myr). This indicated that, once we accounted for divergent adaptive optima for the *M. micrantha* clade and the rest of the phylogeny, the phylogenetic signal became null, and leaf-size evolution around one optimum or the other was not phylogenetically correlated. The same type of observation, but with a greater magnitude, was seen for the evolution of altitudinal niche under the OUM Regime (t_1/2_ = 0.027 Myr) and to the regional niche under the OUM Geo (t_1/2_ = 0.125 Myr) with a reduction of the phylogenetic signal of 33 and 43 times, respectively.

## 4. Discussion

### 4.1. Is the Radiation of Macrocarpaea in the Andes an Adaptive Radiation?

Based on the traits investigated in this study, we found that it is unlikely that the radiation of *Macrocarpaea* in the Andes was associated with niche partitioning (adaptation). Neither the disparification analyses with the MDI statistic nor the model comparison supported the pattern of an “early burst” of disparification expected under adaptive radiation [5,76]. The EB model performed poorly in the model comparisons for all the traits investigated, and its parameter that described the time dependency of trait evolution was always returned null, which collapsed this model to a simple Brownian motion model (BM) penalized for an additional parameter. Despite the purported prevalence of adaptive radiations in nature [14], the early burst pattern of disparification predicted by theory has been inferred only for a relatively small number of study clades [11,12,76] (but see [16]). Recently, it was suggested that the scarcity of evidence for early burst models found to date was probably often the consequence of lack of sufficient statistical power of PCM instead of evidence for the rarity of adaptive radiations in evolution [90]. In our approach, the model comparison was complemented with disparification through time methods that allowed graphical inspection of a trend in the disparification of traits. For most traits investigated, there was no signal of an early burst of disparification, thus confirming the results from the model comparison procedure.

The only potential exception is the latitudinal niche, which reflected the north-to-south climatic variations (principally temperature seasonality) experienced by species occurring in varying regions of the Andes. In the initial history of the clade, the observed disparity curve for the latitudinal niche (not reaching the significance level) was below the median of simulations under the BM process. Vieu et al. [29] proposed that the radiation of *Macrocarpaea* in the Andes was driven by dispersification (diversification associated with dispersal) or range expansion during rapid colonization (<2 Myr) of the MMF biome some 7 Ma. The trajectory of the disparity curve below the median of the BM simulations suggested that, at the time of the burst of species diversification of *Macrocarpaea* in the Andes, latitudinal niche disparity was predominantly partitioned between lineages [76]. It has been recently suggested that climatic niche evolution reflected the history of species migration more than the mode of evolution of their climatic tolerances [91]. Accordingly, if we assume that changes in the latitudinal niche were predominantly associated with dispersal along the Andes, this result is consistent with diversification associated with colonization of previously unoccupied regions. Later, the disparity curve tracks back to the median of the BM simulation, most likely as a consequence of more recent exchanges between the different regions of the Andes.

### 4.2. Pleistocene Climatic Oscillation, an Engine for the Disparity?

We have not found support for a dramatic impact of the Pleistocene climatic oscillations (PCO) on the trait or niche evolution across the Andean *Macrocarpaea* clade as a whole. It is known that the PCO caused dramatic fluctuations in altitudinal distributions of the vegetation in the MMF [35,36]. It has been proposed that this recent dynamic history could have promoted allopatric speciation in the Andes through repeated cycles of species range contraction and re-expansion [37]. The PCO might also have enhanced ecological divergence by driving populations unable to track the pace of the displacement of their habitat to adapt to the new local ecological conditions to survive [92]. In our study, the SHIFT and ER model that specifically account for an influence of the PCO on the tempo and the mode of evolution, respectively [81], performed relatively poorly in comparison with other models explaining *Macrocarpaea* evolution for almost all the traits we investigated. The only exception is the plant size for which the SHIFT and the ER performed substantially better than most of the other models considered but which still did obtain the lower AICc (best fit) in any of the trees from the posterior sample.

It should be noted that the parameter values estimated under the SHIFT and ER models were often biologically unrealistic (Supplementary Materials Table S1). The pre-PCO evolutionary rate (σ_1_) of the SHIFT model is returned null for all the traits, except for the latitudinal niche. Moreover, the selection parameter of the ER model that constrained the evolution around the ancestral optimum for the pre-PCO part of the tree is estimated to be large (α > 146) for leaf size, flower size and altitudinal niche. According to these estimates, no evolution would have occurred in these traits before the PCO, either because of the absence of the evolutionary potential (σ_1_ = 0) or due to strong stabilizing selection pressures (large α). The fossil record documents numerous examples of lineages that experienced trait stasis (retention of the ancestral phenotype) over a million years [93]. However, we think that a complete stasis over almost 4.5 Myr, as suggested here, is unlikely and rather reflects a statistical limitation of models that partition phylogenetic trees into time slices, using PCM. It has been shown through simulations that, in the absence of fossil data, the PCM has a low ability to converge toward the parameter values that generated the simulated data with these types of models [81]. The latitudinal niche is the only trait that has a non-null pre-PCO rate of evolution (σ_1_ = 2.42) under the SHIFT model and a realistic estimate for selection parameter (α = 0.081) under the ER model. Among the traits we investigated, the latitudinal niche displays the stronger phylogenetic signal (Table 4). Thus, in the absence of fossil data, it seems possible that substantial phylogenetic signal could be required to obtain reliable parameter estimates with time-slice models.

### 4.3. Evidence for Elevation Belt, Clade History and Region-Specific Evolution?

We found that the leaf-size evolution was better explained by a model of stabilizing selection that accounted for a divergent adaptive optimum between the *M. micrantha* clade and the rest of the phylogeny (OUM Clade). Plant species with high SLA (thin leaves) tend to have higher photosynthetic rates and shorter leaf life spans and in general characterize fast-growing species in water and nutrient-rich environments [94]. In contrast, plant species with low SLA (thick/dense leaves) tend to have higher nutrient retentions and better protection from desiccation and thus characterize slower-growing species in nutrient and/or water-poor environments. In general, SLA tends to decrease with increasing elevation [95], particularly in the tropics, most likely as a consequence of lower temperatures and lower nutrient availability rather than water deficiency [63]. Concerning the SLA, the OUM Clade model also obtained the best fit, but the model that accounted for divergent optima between the two elevations’ belts (OUM Regime) obtained equivalent support. This was likely because the lower sampling fraction for SLA makes these two models similar and thus statistically hard to discriminate (Supplementary Materials Figure S2). Taken together, the preference of the OUM Clade model for leaf trait evolution indicates that the *M. micrantha* clade escaped from the ancestral adaptive optimum that constrained leaf evolution in the other lineages of *Macrocarpaea* in the Andes and evolved toward a new optimum. These results are consistent with the hypotheses that the colonization of the lower montane forest (LMF < 1800 m) by the *M. micrantha* clade represented a shift into a new adaptive zone. The LMF experiences higher vertical precipitations (rainfall) and higher temperatures than the UMF but also occurs on soils with on average higher nutrient contents [63,96,97,98]. These abiotic factors likely enhance competition for light and thus tend to favor fast-growing species that are often characterized by larger, thinner and shorter-lived leaves than species found in the UMF [99]. Thus, the evolution of larger leaves and the higher SLA in the *M. micrantha* clade likely represents functional adaptations to the LMF conditions that potentially improve the competitive ability of species in this clade in the LMF plant communities.

Interestingly, the few species in the other parts of the phylogeny that occur in the LMF for which we have morphological (four species) and SLA (two species) data do not have especially large leaves nor high SLAs (Figure 5a,d). This likely explains why the OUM Regime that accounts for divergent adaptive optima for the LMF and the UMF obtains only the third-best score to explain leaf size evolution, far behind the OUM Clade model but also the OU1 model that has a single adaptive optimum for the whole phylogeny. One might have expected that natural selection would have led to phenotypic convergence of all the species that occur in the LMF due to similar environmental conditions they experience. Phenotypic convergence has been reported in a wide range of organisms [100,101,102] and is thought to reflect the deterministic nature of evolution on a macroevolutionary adaptive landscape [103]. Here, the phenotypic deviation from the adaptive optimum of the *M. micrantha* clade for the LMF species that do not belong to this clade suggests that (1) either these species were wrongly assigned as occurring in LMF, for example, if the LMF/UMF transition, which is more of a diffused than a strict discontinuity, is located bellow 1800 m where these species occur; (2) or that they do not share the adaptive strategy of the species of the *M. micrantha* clade, possibly because their leaf evolution remains constrained around the ancestral adaptive optimum of *Macrocarpaea* in the Andes. Evolutionary constraints can arise from a variety of processes such as via genetic correlations between several traits (i.e., pleiotropic effects and linkage disequilibrium) but also due to a lack of genetic variation or a limited efficiency of selection compared to the drift in small populations [104,105,106]. Such constraints on leaf evolution might have prevented these *Macrocarpaea* species to respond predictably to the novel selective pressure of the LMF abiotic environment, which, in turn, could have put them at a competitive disadvantage in the LMF plant communities.

The limited niche overlap between the species from the LMF and UMF observed along the altitudinal niche axes (Figure 5e) indicates that the hypothesis of wrong assignments of species to the LMF is plausible. However, it is striking that there was only a single-species divergence event within the LMF outside the *M. micrantha* clade (sampled and non-extinct) in our phylogeny. Whereas we acknowledge that our low sampling fraction of the species from the Northern Andes might exacerbate this pattern, the lack of species diversification we infer in the LMF for lineages outside the *M. micrantha* clade indicates that they have not been particularly successful within the LMF. By comparison, the *M. micrantha* clade forms a relatively large clade of 25 species sampled here, 19 of which occur in the LMF from Northern Ecuador to Central Peru. The study of Vieu et al. [29] detected a potential shift in the diversification rate along the branches, leading to the early diverging part of the *M. micrantha* clade. It is tempting to see a causal link between the switch toward the new adaptive zone and the burst of the species diversification.

The theory of the phenotypic adaptive landscape predicts that the ecological release following the entry into a new adaptive zone should facilitate diversification. Specifically, through the partitioning of the broad adaptive zone into several more specialized sub-zones [64]. In the example of the *M. micrantha* clade, it is not clear whether the potential burst of species diversification has been promoted by finer niche specialization within the LMF. Model comparisons for the evolution of flower size strongly favor the BMM Clade model and the rate of evolution estimated for the *M. micrantha* clade is more than four times higher than the rates estimated for the other lineages. It is perhaps notable that the *M. micrantha* clade includes both the species with the largest and the smallest flowers reported in our phylogeny. These results are consistent with the hypothesis of a release on flower-size evolution associated with the entry of the clade into the LMF. However, it is hard to evaluate how much the faster flower-size evolution is responsible for the burst of species diversification detected at the base of this clade. All species of *Macrocarpaea* have greenish, whitish to yellowish bell-shaped flowers (Figure 1) that are rich in nectar mostly released at night [107] and that are predominantly pollinated by nectar-feeding bats (but are also visited by hummingbirds during the day and moths during the night). In South America, out of the 23 plant-visiting genera of bats (Phyllostomidae), only 10 genera are specialized nectarivores [108]. These bats are generalists and tend to feed on any available nectar sources [109], suggesting that pollinator specialization is an unlikely mechanism driving species divergence in *Macrocarpaea*. This also suggests that diverse *Macrocarpaea* species compete for the same pollinators and the evolution of distinctly sized flowers might have limited interspecific pollen transfer through the spatial segregation of the pollen deposit on bats heads [109]. In the absence of *Macrocarpaea* competitors in the LMF, species of the *M. micrantha* clade might have explored de novo the whole *Macrocarpaea* flower-size spectrum. Thus, pollinator competition is a credible explanation for how flower-size variation could potentially provide a mechanism for prezygotic isolation in *Macrocarpaea* that might translate into species divergence. However, we think that the faster flower-size evolution is unlikely to be the main driver of the pulse of diversification detected at the base of the *micrantha* clade. Instead, the rapid range expansion of the clade that followed its entry in the LMF (Figure 4d; [29]) seems to be a more plausible explanation. In other words, the colonization of the LMF, lacking other *Macrocarpaea* lineages, likely represented an opportunity for a second wave of range expansion associated with species diversification at lower elevations for the *M. micrantha* clade.

Adaptive zone or niche shifts are usually thought to be accompanied or facilitated by the acquisition of a key innovation [64]. A key innovation is often referred to as a new trait that allows the organism to interact with new environments in a novel way (for detailed definitions of key innovation, see [109]). There are several examples in the literature of the acquisition of novel traits that allowed plant lineages to invade a new adaptive zone, such as the cushion life form of the *Androsace* (Primulaceae; [91]) or the pachycaulous stem rosette life form of *Espeletia* (Asteraceae; [110]) that allowed these clades to colonize and diversify in harsh alpine habitats. In the case of the *M. micrantha* clade, no new trait is associated with the adaptation to the LMF, but instead, a change in leaf size, a leaf fundamental trait, seems to have been made possible by the liberation from an ancestral evolutionary constraint. Recently, it has been suggested that constraint breaking mutations are the most prominent agents of biological innovation [111]. Such a constraint breaking mutation(s) could have arisen in the ancestor of the *M. micrantha* clade and been maintained with standing genetic variation. Then, the entry into the LMF through the dispersal of propagules or the fluctuation of the vegetation belts during the PCO might have turned this mutation into an advantageous one that allowed evolution toward a new phenotype adapted to the LMF way of life. The estimated age of the MRCA of the *M. micrantha* clade (2.6 Myr) coincided remarkably closely with the beginning of the PCO. Finally, the fact that a successful entry in the LMF only happened once in *Macrocarpaea* instead of repeatedly along the Andes is consistent with the hypothesis of an advantageous mutation or a set of advantageous mutations unique to the *M. micrantha* clade.

The last trait to be considered is plant size, which broadly describes the varying growth types found in *Macrocarpaea* (small shrubs to tall trees). Model comparisons favored a model that accounted for region-specific rates of evolution (BMM Region) with the rate estimated for the AHZ inferred to be two and four times faster than for the Central Andes and the Northern Andes, respectively. This result supports the hypothesis that the AHZ stimulated morphological (adaptive?) evolution more than any other region of the Andes [65,66]. However, this assertion should be taken with some caution as our low sampling fraction for the Northern Andes might again alter the pattern. Nevertheless, the fact that the *M. micrantha* clade likely originated in the AHZ (Figure 4d; [29]) indicates that the AHZ might have indeed played some important role in the group evolution.

### 4.4. Note on Phylogenetic Half-Life (Phylogenetic Signal)

Our estimation of the phylogenetic signal for the various traits we investigated is in general relatively low. The phylogenetic half-life estimated under the OUM, which represented the amount of time necessary for a clade to move halfway out from the adaptive optimum of its ancestor [79], was about a third of the height of the phylogenetic tree for leaf size, SLA and flower size and about a sixth for the altitudinal niche. This means that phylogenetic relationships are a weak predictor of these traits’ values for a particular species and that closely related species are often more dissimilar than expected under a BM model in regard to their leaf and flower morphologies but also their altitudinal position [91]. The half-life for plant size was substantially greater than for the other morphological traits, suggesting that this particular trait might be more phylogenetically conserved. Concerning the latitudinal niche, the half-life was close to the BM expectation (almost equal to the height of the phylogenetic tree), which indicates that closely related species tend to occur in the same geographical area [29]. However, once we accounted for divergent adaptive optima for each main region the half-life collapsed to a low value, suggesting that, within each region, the geographical position of a species is random with regard to the phylogeny [89]. This means that, whereas closely related species tend to co-occur in the same major region, within each region they tend to be less closely adjacent geographically than expected under a BM model. The same applies to leaf size, SLA and the altitudinal niche. Once we accounted for the divergent adaptive optima, the phylogenetic signal collapsed to low values, indicating that, within the boundaries of an adaptive optimum or an adaptive zone, the position of a particular species is relatively random in regard to the phylogeny. Thus, the phylogenetic signal we measured before accounting for the divergent adaptive optima was likely a measure of the phylogenetic signal for the belonging to particular optima or the other. This implies that, within the boundaries of an adaptive zone, the position of a species is not phylogenetically constrained, but the mere fact of belonging to one adaptive zone or another is. We guard ourselves from interpreting the phylogenetic signal in terms of niche conservatism, as the link between the former and the latter is complex [88,112]. We believe that results from the model comparisons discussed above provide a good idea of the importance of niche conservatism and niche evolution during the diversification of *Macrocarpaea* in the Andes but also of which traits are constrained or not.

The quality of the data we used for the morphological traits might appear as questionable and several individual measures for each species would have been preferable to allow direct estimation of species means and standards deviation. However, we think that the large differences in model fit we obtained with these data suggest that the patterns we infer were relatively robust. Moreover, whereas we tested a relatively wide spectrum of models and specific hypothesis, we acknowledge that this set is not exhaustive and an evolutionary picture with a finer resolution for *Macrocarpaea* in the Andes is possible.

## 5. Conclusions

In this study, we showed that it is unlikely that adaptive divergence fostered the main radiation of *Macrocarpaea* in the MMF. Instead, geographical processes arising from the rapid range expansion of the genus along the Andes, i.e., a pattern of dispersification, are favored. We also showed that, during more than the half of the history of the genus in the Andes, species were restricted almost exclusively to the UMF possibly because of the evolutionary constraints. More recently and coinciding with the beginning of the PCO, the *micrantha* lineage was successfully established and diversified in the LMF. This adaptive zone shift has been accompanied by the evolution of larger and thinner leaves that likely represent adaptations to warmer and wetter conditions in the LMF. We suggest that the development of this new phenotype potentially originated via constraint-breaking mutation(s) acquired by the MRCA of the *M. micrantha* clade. Colonization of the LMF was quickly followed by a new burst of range expansion associated with renewed dispersification along the Andes, corresponding to the secondary burst of diversification detected at the basis of the clade in a previous study [29]. Finally, whereas leaf traits and altitudinal niche variations seem to be bounded within each adaptive zone, variation within these bounds is relatively random in regard to the phylogeny. Overall, the results we report here are in close agreement with the theory of quantum evolution of Simpson [64] that predicts that evolution is relatively stable and bounded during relatively long periods, but that occasionally adaptive transitions occur.

## Figures and Tables

**Table 1:** Loadings, eigenvalues and variance explained by the two first-principal components from an analysis of 5 morphological traits for 55 Andean *Macrocarpaea* species. Results displayed are from the phylogenetic PCA based on the MCC tree.

	pPC1	pPC2
Blade length	−0.96	0.08
Blade width	−0.97	0.09
Calyx width	−0.40	−0.78
Corolla length	−0.28	−0.65
Corolla width	−0.11	−0.89
Eigenvalue	0.19	0.05
Proportion of Variance	0.70	0.18
Cumulative proportion	0.70	0.88

**Table 2:** Loadings, eigenvalues and variance explained by the two firsts principal components from an analysis of the 19 BioClim variables together with 3 geo-position variables for 62 Andean *Macrocarpaea* species. Results displayed are from the phylogenetic PCA based on the MCC tree. Variables are as follows: LAT = latitude, LONG = longitude, ALT = altitude, BIO1 = annual mean temperature; BIO2 = mean diurnal temperature range [mean of monthly (maximum temperature) minimum temperature)]; BIO3 = isothermality (BIO2/BIO7 × 100); BIO4 = temperature seasonality (standard deviation of monthly temperature); BIO5 = maximum temperature of the warmest month; BIO6 = minimum temperature of the coldest month; BIO7 = temperature range (BIO6–BIO5); BIO8 = mean temperature of the wettest quarter; BIO9 = mean temperature of the driest quarter; BIO10 = mean temperature of the warmest quarter; BIO11 = mean temperature of the coldest quarter; BIO12 = annual precipitation; BIO13 = precipitation of the wettest month; BIO14 = precipitation of the driest month; BIO15 = precipitation seasonality (standard deviation of monthly precipitation); BIO16 = precipitation of the wettest quarter; BIO17 = precipitation of the driest quarter; BIO18 = precipitation of the warmest quarter; BIO19 = precipitation of the coldest quarter.

	pPC1	pPC2
LAT	0.06	0.88
LONG	0.06	−0.82
ALT	−0.92	0.11
BIO1	0.89	−0.19
BIO2	−0.48	−0.54
BIO3	−0.03	0.69
BIO4	0.02	−0.82
BIO5	0.86	−0.33
BIO6	0.92	0.09
BIO7	−0.41	−0.77
BIO8	0.88	−0.23
BIO9	0.92	−0.08
BIO10	0.90	−0.24
BIO11	0.89	−0.10
BIO12	0.82	0.07
BIO13	0.71	−0.14
BIO14	0.71	0.26
BIO15	−0.36	−0.46
BIO16	0.71	−0.15
BIO17	0.72	0.26
BIO18	0.70	−0.14
BIO19	0.68	0.30
Eigenvalue	10.67	4.27
Proportion of Variance	0.48	0.19
Cumulative proportion	0.48	0.68

**Table 3:** Results from the model comparison analyses of the evolution of the 6 traits investigated in the study. Values displayed are ΔAICc estimated from the MCC tree together with the 10%, 50% and 90% quantiles (in parenthesis) of the distribution of ΔAICc estimated from the posterior distribution of trees (100 trees). We ask the reader to refer to the main text for a detailed explanation of the 11 models compared. For each trait, the model(s) that obtain the best fit is (are) in bold.

Model	Leaf Size	Flower Size	Plant Size	SLA	Altitudinal Niche	Latitudinal Niche
BM1	14.25 (8.13;15.36;23.30)	13.97 (5.96;12.61;17.78)	4.40 (3.23;5.04;7.20)	9.99 (5.72;8.34;20.27)	77.93 (63.48;77.38;100.04)	79.40 (60.24;80.58;91.53)
EB	16.49 (10.37;17.60;25.54)	16.21 (8.20;14.85;20.02)	6.64 (5.47;7.28;9.44)	12.45 (8.18;10.80;22.73)	80.14 (65.69;79.59;102.26)	81.61 (62.45;82.79;93.74)
OU1	7.66 (0.00;7.68;11.42)	7.03 (0.50;5.31;9.07)	6.73 (4.31;6.07;7.85)	9.17 (6.37;8.06;11.78)	59.96 (55.68;59.31;63.19)	82.57 (58.71;82.73;93.85)
SHIFT	9.60 (3.27;11.13;16.00)	9.06 (2.09;7.81;11.88)	2.16 (0.97;2.94;5.16)	9.36 (5.17;7.46;17.95)	70.22 (57.76;69.77;90.41)	81.17 (60.62;82.01;92.99)
ER	9.378 (2.95;10.76;15.97)	9.06 (1.36;7.82;11.96)	1.84 (0.73;2.59;4.33)	8.75 (4.90;7.01;17.72)	70.22 (57.76;69.77;90.41)	81.47 (61.31;82.60;93.43)
BMM Regime	14.01 (7.16;14.42;20.22)	14.37 (4.88;13.31;17.19)	5.58 (4.51;6.35;8.81)	10.66 (7.02;9.51;15.76)	58.95 (51.94;61.23;74.03)	76.40 (59.06;78.32;88.14)
OUM Regime	9.04 (1.22;8.60;12.62)	9.29 (2.74;7.45;11.20)	9.06 (6.61;8.39;10.07)	1.74 (0.00;0.00;4.28)	0.00 (0.00;0.00;0.00)	81.46 (57.20;80.35;91.47)
BMM Clade	15.77 (8.04;15.69;22.33)	0.00 (0.00;0.00;1.05)	3.74 (3.16;6.39;8.67)	11.46 (7.71;10.15;17.33)	80.13 (63.68;79.59;101.79)	81.60 (59.39;81.21;92.58)
OUM Clade	0.00 (0.00;0.00;1.72)	9.36 (0.69;7.48;11.19)	8.59 (6.35;8.12;9.80)	0.00 (0.00;0.091;9.24)	50.92 (43.44;50.43;58.57)	84.80 (60.10;84.43;95.96)
BMM Geo	9.99 (2.31;10.06;16.67)	14.45 (6.80;12.38;19.10)	0.00 (0.00;0.00;0.00)	12.35 (8.10;10.63;21.75)	76.03 (65.59;75.68;94.45)	33.94 (30.36;42.10;57.67)
OUM Geo	11.28 (3.36;11.24;14.90)	10.13 (3.33;8.11;11.80)	7.04 (4.32;6.37;8.47)	11.80 (8.93;10.70;14.43)	60.40 (56.80;59.81;63.16)	0.00 (0.00;0.00;0.00)

**Table 4:** Phylogenetic half-life and stationary variance of the 6 traits considered in the study estimated under the evolutionary model OU1 and OUM if for a particular trait an OUM model obtained the best fit. For leaf size, the OUM is the OUM Clade. For SLA and altitudinal niche, the OUM is the OUM Regime. For the latitudinal niche, the OUM is the OUM Geo. The values displayed are estimated from the MCC tree together within parenthesis the 10%, 50% and 90% quantiles of the distribution of ΔAICc estimated from the posterior distribution of trees (100 trees). The phylogenetic half-life (t_1/2_ = ln2/α) is the time in millions of years (Ma) taken for an OU process to erase half the phylogenetic covariance between sister taxa. A half-life equal to the age of the MRCA of a clade (here, 7.2 Ma) indicates a phylogenetic signal equivalent to the expectation under a BM model of evolution (strong signal), whereas a half-life smaller than the age of the youngest node in a clade (here 0.36 Ma) indicates no phylogenetic correlations (no signal). The stationary variance (*V_y_ =* σ/2*α) quantifies the relative strength of drift and directional selection around an adaptive optimum.

Trait	Half-Life OU1	Stationary Variance OU1	Half-Life OUM	Stationary Variance OUM
Leaf size	1.938 (1.294;1.635;2.415)	0.066 (0.043;0.094;0.151)	0.312 (0.054;0.266;1.47)	1.69 (0.106;2.389;56.093)
Flower size	1.797 (1.340;1.729;2.359)	0.019 (0.011;0.021;0.034)	NA	NA
Plant Size	3.658 (2.266;2.925;4.385)	0.009 (0.006;0.013;0.021)	NA	NA
SLA	1.951 (1.117;1.978;2.557)	0.028 (0.017;0.028;0.089)	0.022 (0.014;0.888;1.828)	123.654 (0.026;0.094;316.945)
Altitudinal niche	1.011 (0.086;0.920;1.497)	9.483 (5.454;10.156;769.061)	0.027 (0.008;0.020;0.194)	4676.83 (88.81;8948.43;39845.79)
Latitudinal niche	6.758 (2.764;5.435;8.854)	0.279 (0.195;0.368;0.858)	0.139 (0.033;0.125;0.563)	64.658 (4.228;81.014;1139.458)

## Data Availability

Additional datasets generated and analyzed in this study that are not presented here or in the Supplementary Materials are available upon request from the corresponding author.

## Supplementary Materials

The following are available online at https://www.mdpi.com/article/10.3390/biology10090825/s1. Figure S1: Mapping of time slices (a), regimes (b), taxonomy (c) and geography (d) on the MCC tree of the Andean species of *Macrocarpaea* for which climatic data were available (62 species). See Figure 4 caption for further details. Figure S2: Mapping of time slices (a), regimes (b), taxonomy (c) and geography (d) on the MCC tree of the Andean species of Macrocarpaea that have specific leaf area (SLA) data (31 species). See Figure 4 caption for further details. Figure S3: Distributions of the likelihood ratio statistic (δ) for the two best-fitting models for each of the 6 traits investigated in the study. In each case, the lighter distribution shows the distribution of δ values obtained by bootstrapping under the less fit of the two models, whereas the darker distribution shows the distribution under the best fitting model. A total of 2000 replicates are used for each distribution. The dashed vertical line indicates the observed value of δ when the models are fit to the real dataset. The models compared for each trait are indicated in the legend at the top right or the top left corner of individual figures. Table S1: Results from the evolutionary model comparison for the six traits investigated in the study and corresponding parameter estimates. The values displayed are estimated on the MCC tree together within parenthesis, the 10%, 50%, and 90% quantiles of the distribution estimated from the posterior distribution trees (100 trees). X0 is the estimated root value under BM1, SHIFT and BMM models. The θ1 is the unique optimum of the OU1 and the ER model or the first optimum of the OUM models. The σ1 is the unique evolutionary rate of the BM1, ER and OUM models or the first evolutionary rate of the SHIFT and BMM models. The r is the variation through time parameter of the EB model. The α is the selection strength parameter of the OU1, ER and OUM models. The σ2 and σ3 are the second and third drifting rate parameters of the SHIFT and BMM models. The θ2 and θ3 are the second and third adaptive optima of the OUM models. NA indicates that the corresponding parameter is not included in the model.

## Abbreviations

AHZ	Amotape-Huancabamba zone
AICc	Akaike information criterion
BioClim	bioclimatic
BM	Brownian motion model of evolution
BMM Regime	two-rate BM model
BMM Geo	three rate BM model
DTT	disparity through time
EB	Early burst model
ER	ecological release model
LMF	lower montane forest
Ma	Million years ago
MCC tree	maximum clade credibility tree
MDI	morphological disparity index
MMF	middle elevation montane forest
MRCA	most recent common ancestor
Myr	million years
NRMSE	normalized root squared error
OU	Ornstein–Uhlenbeck model with a single optimum
OUM Geo	OU model with three optima
OUM Regime	OU model with two optima
PIC	phylogenetic independent contrasts
PCM	phylogenetic comparative method
PCO	Pleistocene climatic oscillations
pPCA	phylogenetic principal components analysis
SHIFT	a two-rate BM model
SLA	specific leaf area
UMF	upper montane forest

## References

[1] Mahler D.L., Revell L.J., Glor R.E., Losos J.B. (2010). Ecological Opportunity and the Rate of Morphological Evolution in the Diversification of Greater Antillean Anoles. Evol. Int. J. Org. Evol..

[2] Losos J.B. (2010). Adaptive Radiation, Ecological Opportunity, and Evolutionary Determinism. American Society of Naturalists E. O. Wilson Award Address. Am. Nat..

[3] Yoder J.B., Clancey E., Des Roches S., Eastman J.M., Gentry L., Godsoe W., Hagey T.J., Jochimsen D., Oswald B.P., Robertson J. (2010). Ecological Opportunity and the Origin of Adaptive Radiations. J. Evol. Biol..

[4] Losos J.B., Mahler D.L., Bell M.A., Futuyma D.J., Eanes W.F., Levinton J.S. (2010). Adaptive Radiation: The Interaction of Ecological Opportunity, Adaptation, and Speciation. Evolution Since Darwin: The First 150 Years.

[5] Gavrilets S., Losos J.B. (2009). Adaptive Radiation: Contrasting Theory with Data. Science.

[6] Grant P.R., Grant B.R. (2006). Evolution of Character Displacement in Darwin’s Finches. Science.

[7] Losos J.B., Jackman T.R., Arson A., Queiroz K., Rodriguez-Schettino L. (1998). Contingency and Determinism in Replicated Adaptive Radiations of Island Lizards. Science.

[8] Seehausen O. (2006). African Cichlid Fish: A Model System in Adaptive Radiation Research. Proc. R. Soc. B Biol. Sci..

[9] Robichaux R.H., Carr G.D., Liebman M., Pearcy R.W. (1990). Adaptive Radiation of the Hawaiian Silversword Alliance (Compositae-Madiinae): Ecological, Morphological and Physiological Diversity. Ann. Mo. Bot. Gard..

[10] Burbrink F.T., Pyron R.A. (2010). How Does Ecological Opportunity Influence Rates of Speciation, Extinction, and Morphological Diversification in New World Ratsnakes (Tribe Lampropeltini)?. Evol. Int. J. Org. Evol..

[11] Schweizer M., Hertwig S.T., Seehausen O. (2014). Diversity versus Disparity and the Role of Ecological Opportunity in a Continental Bird Radiation. J. Biogeogr..

[12] Slater G.J., Price S.A., Santini F., Alfaro M.E. (2010). Diversity versus Disparity and the Radiation of Modern Cetaceans. Proc. R. Soc. B Biol. Sci..

[13] Benkman C.W. (2003). Divergent Selection Drives the Adaptive Radiation of Crossbills. Evol. Int. J. Org. Evol..

[14] Schluter D. (2000). The Ecology of Adaptive Radiation (Oxford Series in Ecology and Evolution).

[15] Adams D.C., Berns C.M., Kozak K.H., Wiens J.J. (2009). Are Rates of Species Diversification Correlated with Rates of Morphological Evolution?. Proc. R. Soc. B Biol. Sci..

[16] Harmon L.J., Losos J.B., Jonathan Davies T., Gillespie R.G., Gittleman J.L., Bryan Jennings W., Kozak K.H., McPeek M.A., Moreno-Roark F., Near T.J. (2010). Early Bursts of Body Size and Shape Evolution Are Rare in Comparative Data. Evol. Int. J. Org. Evol..

[17] Burbrink F.T., Chen X., Myers E.A., Brandley M.C., Pyron R.A. (2012). Evidence for Determinism in Species Diversification and Contingency in Phenotypic Evolution during Adaptive Radiation. Proc. R. Soc. B Biol. Sci..

[18] Hipsley C.A., Miles D.B., Müller J. (2014). Morphological Disparity Opposes Latitudinal Diversity Gradient in Lacertid Lizards. Biol. Lett..

[19] Rabosky D.L., Donnellan S.C., Grundler M., Lovette I.J. (2014). Analysis and Visualization of Complex Macroevolutionary Dynamics: An Example from Australian Scincid Lizards. Syst. Biol..

[20] Rowe K.C., Aplin K.P., Baverstock P.R., Moritz C. (2011). Recent and Rapid Speciation with Limited Morphological Disparity in the Genus Rattus. Syst. Biol..

[21] Rundell R.J., Price T.D. (2009). Adaptive Radiation, Nonadaptive Radiation, Ecological Speciation and Nonecological Speciation. Trends Ecol. Evol..

[22] Wiens J.J. (2004). Speciation and Ecology Revisited: Phylogenetic Niche Conservatism and the Origin of Species. Evolution.

[23] Gittenberger E. (1991). What about Non-Adaptive Radiation?. Biol. J. Linn. Soc..

[24] Kozak K.H., Weisrock D.W., Larson A. (2006). Rapid Lineage Accumulation in a Non-Adaptive Radiation: Phylogenetic Analysis of Diversification Rates in Eastern North American Woodland Salamanders (Plethodontidae: Plethodon). Proc. R. Soc. B Biol. Sci..

[25] Wellenreuther M., Sánchez-Guillén R.A. (2016). Nonadaptive Radiation in Damselflies. Evol. Appl..

[26] Lambert J.W., Reichard M., Pincheira-Donoso D. (2019). Live Fast, Diversify Non-Adaptively: Evolutionary Diversification of Exceptionally Short-Lived Annual Killifishes. BMC Evol. Biol..

[27] Comes H.P., Tribsch A., Bittkau C. (2008). Plant Speciation in Continental Island Floras as Exemplified by *Nigella* in the Aegean Archipelago. Philos. Trans. R. Soc. Lond. B. Biol. Sci..

[28] Orme C.D.L., Davies R.G., Burgess M., Eigenbrod F., Pickup N., Olson V.A., Webster A.J., Ding T.-S., Rasmussen P.C., Ridgely R.S. (2005). Global Hotspots of Species Richness Are Not Congruent with Endemism or Threat. Nature.

[29] Vieu J.C., Hughes C.E., Kissling J., Grant J.R. (2021). Evolutionary Diversification in the Hyper-Diverse Montane Forests of the Tropical Andes: The Radiation of the Plant Genus *Macrocarpaea* (Gentianaceae) and the Possible Role of Range Expansion. Bot. J. Linn. Soc..

[30] Doebeli M., Dieckmann U. (2003). Speciation along Environmental Gradients. Nature.

[31] Coyne J.A. (1992). Genetics and Speciation. Nature.

[32] Hoorn C., Wesselingh F.P., ter Steege H., Bermudez M.A., Mora A., Sevink J., Sanmartín I., Sanchez-Meseguer A., Anderson C.L., Figueiredo J.P. (2010). Amazonia Through Time: Andean Uplift, Climate Change, Landscape Evolution, and Biodiversity. Science.

[33] Insel N., Poulsen C.J., Ehlers T.A., Sturm C. (2012). Response of Meteoric Δ18O to Surface Uplift—Implications for Cenozoic Andean Plateau Growth. Earth Planet. Sci. Lett..

[34] van der Hammen T., Hooghiemstra H. (2000). Neogene and Quaternary History of Vegetation, Climate, and Plant Diversity in Amazonia. Quat. Sci. Rev..

[35] Flantua S.G.A., Hooghiemstra H., van Boxel J.H., Cabrera M., González-Carranza Z., González-Arango C. (2014). Connectivity Dynamics Since the Last Glacial Maximum in the Northern Andes: A Pollen-Driven Framework to Assess Potential Migration.

[36] Hooghiemstra H., Van der Hammen T. (2004). Quaternary Ice-Age Dynamics in the Colombian Andes: Developing an Understanding of Our Legacy. Philos. Trans. R. Soc. B Biol. Sci..

[37] Rull V. (2011). Neotropical Biodiversity: Timing and Potential Drivers. Trends Ecol. Evol..

[38] Elias M., Joron M., Willmott K., Silva-Brandão K.L., Kaiser V., Arias C.F., Gomez Piñerez L.M., Uribe S., Brower A.V.Z., Freitas A.V.L. (2009). Out of the Andes: Patterns of Diversification in Clearwing Butterflies. Mol. Ecol..

[39] Benham P.M., Cuervo A.M., McGuire J.A., Witt C.C. (2014). Biogeography of the Andean Metaltail Hummingbirds: Contrasting Evolutionary Histories of Tree Line and Habitat-Generalist Clades. J. Biogeogr..

[40] Gutiérrez-Pinto N., Cuervo A.M., Miranda J., Pérez-Emán J.L., Brumfield R.T., Cadena C.D. (2012). Non-Monophyly and Deep Genetic Differentiation across Low-Elevation Barriers in a Neotropical Montane Bird (*Basileuterus Tristriatus*; Aves: Parulidae). Mol. Phylogenet. Evol..

[41] Casner K.L., Pyrcz T.W. (2010). Patterns and Timing of Diversification in a Tropical Montane Butterfly Genus, *Lymanopoda* (Nymphalidae, Satyrinae). Ecography.

[42] Hutter C.R., Guayasamin J.M., Wiens J.J. (2013). Explaining Andean Megadiversity: The Evolutionary and Ecological Causes of Glassfrog Elevational Richness Patterns. Ecol. Lett..

[43] Patton J.L., Smith M.F. (1992). MtDNA Phylogeny of Andean Mice: A Test of Diversification across Ecological Gradients. Evolution.

[44] Antonelli A., Sanmartín I. (2011). Mass Extinction, Gradual Cooling, or Rapid Radiation? Reconstructing the Spatiotemporal Evolution of the Ancient Angiosperm Genus *Hedyosmum* (Chloranthaceae) Using Empirical and Simulated Approaches. Syst. Biol..

[45] Givnish T.J., Barfuss M.H.J., Ee B.V., Riina R., Schulte K., Horres R., Gonsiska P.A., Jabaily R.S., Crayn D.M., Smith J.A.C. (2014). Adaptive Radiation, Correlated and Contingent Evolution, and Net Species Diversification in Bromeliaceae. Mol. Phylogenet. Evol..

[46] Lagomarsino L.P., Condamine F.L., Antonelli A., Mulch A., Davis C.C. (2016). The Abiotic and Biotic Drivers of Rapid Diversification in Andean Bellflowers (Campanulaceae). New Phytol..

[47] Spriggs E.L., Clement W.L., Sweeney P.W., Madriñán S., Edwards E.J., Donoghue M.J. (2015). Temperate Radiations and Dying Embers of a Tropical Past: The Diversification of Viburnum. New Phytol..

[48] Grant J.R., Rybczyński J.J., Davey M.R., Mikuła A. (2014). A monographic revision of the neotropical genus *Macrocarpaea* (Gentianaceae) in Ecuador. The Gentianaceae—Volume 1: Characterization and Ecology.

[49] Grant J.R. (2014). *De Macrocarpaeae Grisebach (Ex Gentianaceis) Speciebus Novis* XI: Five New Species from the Andes of Ecuador and Colombia. Harv. Pap. Bot..

[50] Grant J.R. (2011). *De Macrocarpaeae Grisebach (Ex Gentianaceis) Speciebus Novis* IX: A Synopsis of the Genus in Bolivia. Harv. Pap. Bot..

[51] Grant J.R. (2008). *De Macrocarpaeae Grisebach (Ex Gentianaceis) Speciebus Novis* VIII: Two New Species from Ecuador. Harv. Pap. Bot..

[52] Grant J.R. (2007). *De Macrocarpaeae Grisebach (Ex Gentianaceis) Speciebus Novis* VII: Four New Species and Two Natural Hybrids. Harv. Pap. Bot..

[53] Grant J.R. (2005). *De Macrocarpaeae Grisebach (Ex Gentianaceis) Speciebus Novis* VI: Seed Morphology, Palynology, an Infrageneric Classification, and Another Twenty-Three New Species, Largely from Colombia. Harv. Pap..

[54] Grant J.R. (2004). *De Macrocarpaeae Grisebach (Ex Gentianaceis) Speciebus Novis* V: Twenty-Three New Species Largely from Peru, and Typification of All Species in the Genus. Harv. Pap. Bot..

[55] Grant J.R. (2003). De Macrocarpaeae Grisebach (Ex Gentianaceis) Speciebus Novis II: Typification of the Ruiz & Pavon Names. Harv. Pap. Bot..

[56] Grant J.R., Struwe L. (2003). *De Macrocarpaeae Grisebach (Ex Gentianaceis) Speciebus Novis* III: Six New Species of Moon-Gentians (*Macrocarpaea*, Gentianaceae: Helieae) from Parque Nacional Podocarpus, Ecuador. Harv. Pap. Bot..

[57] Grant J.R., Struwe L. (2001). *De Macrocarpaeae Grisebach (Ex Gentianaceis) Speciebus Novis* I: An Introduction to the Genus *Macrocarpaea* and Three New Species from Colombia, Ecuador, and Guyana. Harv. Pap. Bot..

[58] Grant J.R., Trunz V. (2011). *De Macrocarpaeae Grisebach (Ex Gentianaceis) Speciebus Novis* X: A Synopsis of the Genus in Montane Atlantic Forests of Brazil. Harv. Pap. Bot..

[59] Grant J.R., Vieu J. (2014). *De Macrocarpaeae Grisebach (Ex Gentianaceis) Speciebus Novis* XII: Three New Species from the Andes of Peru. Harv. Pap. Bot..

[60] Grant J.R., Weaver R.E. (2003). *De Macrocarpaeae Grisebach (Ex Gentianaceis) Speciebus Novis* IV: Twelve New Species of *Macrocarpaea* (Gentianaceae: Helieae) from Central and South America, and the First Report of the Presence of a Stipule in the Family. Harv. Pap. Bot..

[61] Struwe L., Haag S., Heiberg E., Grant J.R. (2009). Andean Speciation and Vicariance in Neotropical *Macrocarpaea* (Gentianaceae-Helieae). Ann. Mo. Bot. Gard..

[62] Struwe L., Albert V.A., Calió F.M., Frasier C., Lepis K.B., Mathews K.G., Grant J.R. (2009). Evolutionary Patterns in Neotropical Helieae (Gentianaceae): Evidence from Morphology, Chloroplast and Nuclear DNA Sequences. Taxon.

[63] Leuschner C., Moser G. (2008). Carbon allocation and productivity in tropical mountain forests. Tropical Mountain Forest: Patterns and Processes in a Biodiversity Hotspot.

[64] Simpson G.G. (1953). The Major Features of Evolution.

[65] Weigend M. (2002). Observations on the Biogeography of the Amotape-Huancabamba Zone in Northern Peru. Bot. Rev..

[66] Mutke J., Jacobs R., Meyers K., Henning T., Weigend M. (2014). Diversity Patterns of Selected Andean Plant Groups Correspond to Topography and Habitat Dynamics, Not Orogeny. Front. Genet..

[67] Penone C., Davidson A.D., Shoemaker K.T., Marco M.D., Rondinini C., Brooks T.M., Young B.E., Graham C.H., Costa G.C. (2014). Imputation of Missing Data in Life-History Trait Datasets: Which Approach Performs the Best?. Methods Ecol. Evol..

[68] Santos T. Package “PVR”. https://CRAN.R-project.org/package=PVR.

[69] Team R.C. (2014). R: A Language and Environment for Statistical Computing.

[70] Stekhoven D.J., Buehlmann P. (2012). MissForest—Non-Parametric Missing Value Imputation for Mixed-Type Data. Bioinformatics.

[71] Revell L.J. (2012). Phytools: An R Package for Phylogenetic Comparative Biology (and Other Things). Methods Ecol. Evol..

[72] Revell L.J. (2009). Size-Correction and Principal Components for Interspecific Comparative Studies. Evolution.

[73] Hijmans R.J., Cameron S.E., Parra J.L., Jones P.G., Jarvis A. (2005). Very High Resolution Interpolated Climate Surfaces for Global Land Areas. Int. J. Climatol..

[74] Hijmans R.J., van Etten J., Mattiuzzi M., Hijmans M.R.J. Package “Raster”. https://cran.r-project.org/web/packages/raster/index.html.

[75] Wright I.J., Reich P.B., Westoby M., Ackerly D.D., Baruch Z., Bongers F., Cavender-Bares J., Chapin T., Cornelissen J.H.C., Diemer M. (2004). The Worldwide Leaf Economics Spectrum. Nature.

[76] Harmon L.J., Schulte J.A., Larson A., Losos J.B. (2003). Tempo and Mode of Evolutionary Radiation in Iguanian Lizards. Science.

[77] Harmon L.J., Weir J.T., Brock C.D., Glor R.E., Challenger W. (2008). GEIGER: Investigating Evolutionary Radiations. Bioinformatics.

[78] Butler M.A., King A.A. (2004). Phylogenetic Comparative Analysis: A Modeling Approach for Adaptive Evolution. Am. Nat..

[79] Hansen T.F. (1997). Stabilizing Selection and the Comparative Analysis of Adaptation. Evolution.

[80] O’Meara B.C., Ané C., Sanderson M.J., Wainwright P.C. (2006). Testing for Different Rates of Continuous Trait Evolution Using Likelihood. Evol. Int. J. Org. Evol..

[81] Slater G.J. (2013). Phylogenetic Evidence for a Shift in the Mode of Mammalian Body Size Evolution at the Cretaceous-Palaeogene Boundary. Methods Ecol. Evol..

[82] Clavel J., Escarguel G., Merceron G. (2015). Mvmorph: An r Package for Fitting Multivariate Evolutionary Models to Morphometric Data. Methods Ecol. Evol..

[83] Boettiger C., Coop G., Ralph P. (2012). Is Your Phylogeny Informative? Measuring the Power of Comparative Methods. Evolution.

[84] Beaulieu J.M., O’Meara B.C. (2012). OUwie: Analysis of Evolutionary Rates in an OU Framework. R Package Version.

[85] Grandcolas P., Nattier R., Legendre F., Pellens R. (2011). Mapping Extrinsic Traits Such as Extinction Risks or Modelled Bioclimatic Niches on Phylogenies: Does It Make Sense at All?. Cladistics.

[86] Burnham K.P., Anderson D.R. (2002). Model Selection and Multimodel Inference: A Practical Information-Theoretic Approach.

[87] Hansen T.F., Garamszegi L.Z. (2014). Use and Misuse of Comparative Methods in the Study of Adaptation. Modern Phylogenetic Comparative Methods and Their Application in Evolutionary Biology: Concepts and Practice.

[88] Münkemüller T., Boucher F.C., Thuiller W., Lavergne S. (2015). Phylogenetic Niche Conservatism—Common Pitfalls and Ways Forward. Funct. Ecol..

[89] Uyeda J.C., Harmon L.J. (2014). A Novel Bayesian Method for Inferring and Interpreting the Dynamics of Adaptive Landscapes from Phylogenetic Comparative Data. Syst. Biol..

[90] Slater G.J., Pennell M.W. (2014). Robust Regression and Posterior Predictive Simulation Increase Power to Detect Early Bursts of Trait Evolution. Syst. Biol..

[91] Boucher F.C., Thuiller W., Davies T.J., Lavergne S. (2014). Neutral Biogeography and the Evolution of Climatic Niches. Am. Nat..

[92] Donoghue M.J. (2008). A Phylogenetic Perspective on the Distribution of Plant Diversity. Proc. Natl. Acad. Sci. USA.

[93] Eldredge N., Thompson J.N., Brakefield P.M., Gavrilets S., Jablonski D., Jackson J.B.C., Lenski R.E., Lieberman B.S., McPeek M.A., Miller W. (2005). The Dynamics of Evolutionary Stasis. Paleobiology.

[94] Reich P.B., Walters M.B., Ellsworth D.S. (1997). From Tropics to Tundra: Global Convergence in Plant Functioning. Proc. Natl. Acad. Sci. USA.

[95] Scheepens J.F., Frei E.S., Stöcklin J. (2010). Genotypic and Environmental Variation in Specific Leaf Area in a Widespread Alpine Plant after Transplantation to Different Altitudes. Oecologia.

[96] Webster G.L., Churchill S.P., Balslev H., Forero E., Luteyn J.L. (1995). The panorama of Neotropical cloud forests. Biodiversity and Conservation of Neotropical Montane Forests: Proceedings of Neotropical Montane Forest Biodiversity and Conservation Symposium.

[97] Beck E., Richter M., Gradstein S.R., Homeier J., Gansert D. (2008). Ecological aspects of a biodiversity hotspot in the Andes of southern Ecuador. The Tropical Mountain Forest: Patterns and Processes in a Biodiversity Hotspot.

[98] Gerold G., Gradstein S.R., Homeier J., Gansert D. (2008). Soil, climate and vegetation of tropical montane forests—A case study from the Yungas, Bolivia. Tropical Mountain Forest: Patterns and Processes in a Biodiversity Hotspot.

[99] Moser G., Hertel D., Leuschner C. (2007). Altitudinal Change in LAI and Stand Leaf Biomass in Tropical Montane Forests: A Transect Study in Ecuador and a Pan-Tropical Meta-Analysis. Ecosystems.

[100] Pupo G.M., Lan R., Reeves P.R. (2000). Multiple Independent Origins of Shigella Clones of *Escherichia Coli* and Convergent Evolution of Many of Their Characteristics. Proc. Natl. Acad. Sci. USA.

[101] Losos J.B. (2011). Convergence, Adaptation, and Constraint. Evol. Int. J. Org. Evol..

[102] Frédérich B., Sorenson L., Santini F., Slater G.J., Alfaro M.E., Heard A.E.S.B., Day E.T. (2013). Iterative Ecological Radiation and Convergence during the Evolutionary History of Damselfishes (Pomacentridae). Am. Nat..

[103] Mahler D.L., Ingram T., Revell L.J., Losos J.B. (2013). Exceptional convergence on the macroevolutionary landscape in island lizard radiations. Science.

[104] Arnold S.J. (1992). Constraints on Phenotypic Evolution. Am. Nat..

[105] Agrawal A.F., Stinchcombe J.R. (2009). How Much Do Genetic Covariances Alter the Rate of Adaptation?. Proc. R. Soc. B Biol. Sci..

[106] Futuyma D.J. (2010). Evolutionary Constraint and Ecological Consequences. Evolution.

[107] Wolff D. (2006). Nectar Sugar Composition and Volumes of 47 Species of Gentianales from a Southern Ecuadorian Montane Forest. Ann. Bot..

[108] Fleming T.H., Geiselman C., Kress W.J. (2009). The Evolution of Bat Pollination: A Phylogenetic Perspective. Ann. Bot..

[109] Muchhala N. (2008). Functional Significance of Interspecific Variation in *Burmeistera* Flower Morphology: Evidence from Nectar Bat Captures in Ecuador. Biotropica.

[110] Monasterio M., Sarmiento L. (1991). Adaptive Radiation of Espeletia in the Cold Andean Tropics. Trends Ecol. Evol..

[111] Nei M. (2013). Mutation-Driven Evolution.

[112] Revell L.J., Harmon L.J., Collar D.C. (2008). Phylogenetic Signal, Evolutionary Process, and Rate. Syst. Biol..

