# Supplementary figures and images for: The Evolution of Trait Disparity during the Radiation of the Plant Genus Macrocarpaea (Gentianaceae) in the Tropical Andes
Source: Biology (Basel). 2021 Aug 25;10(9):825. doi: 10.3390/biology10090825 (PMC8470149; doi:10.3390/biology10090825)

**a)**SHIFT  
&  
ER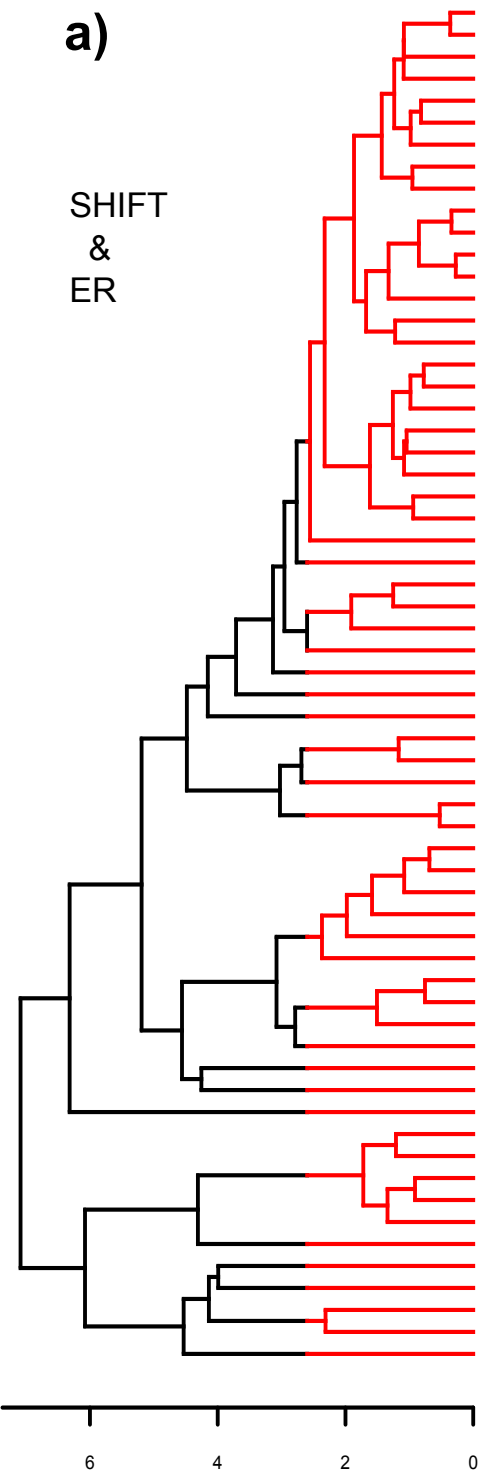**b)**BMM Regime  
&  
OUM Regime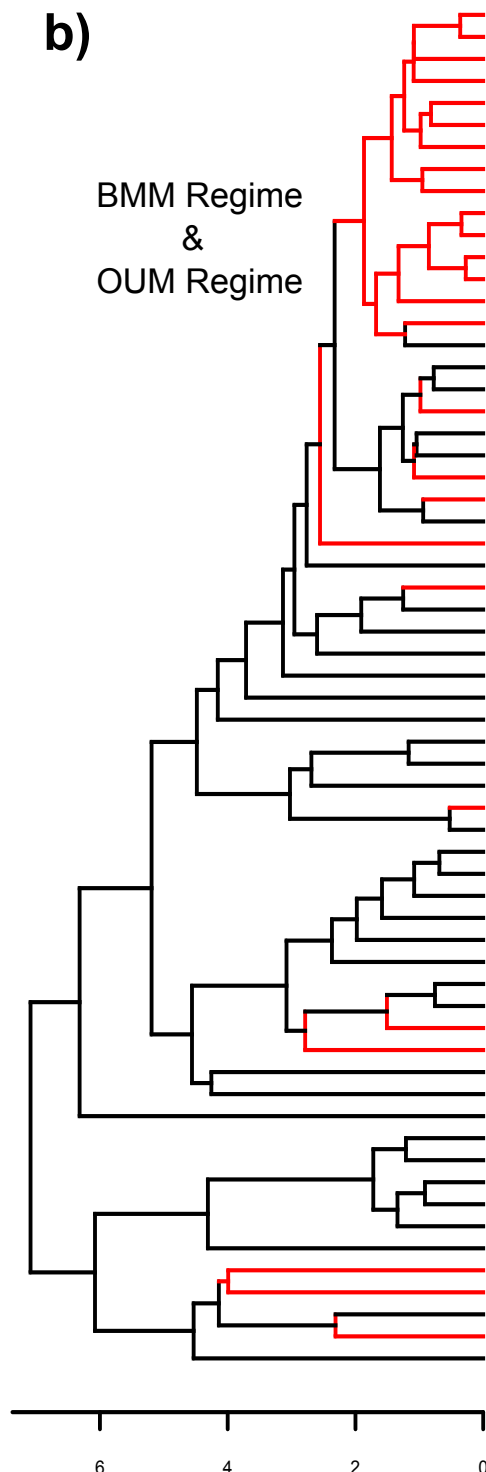**c)**BMM Clade  
&  
OUM Clade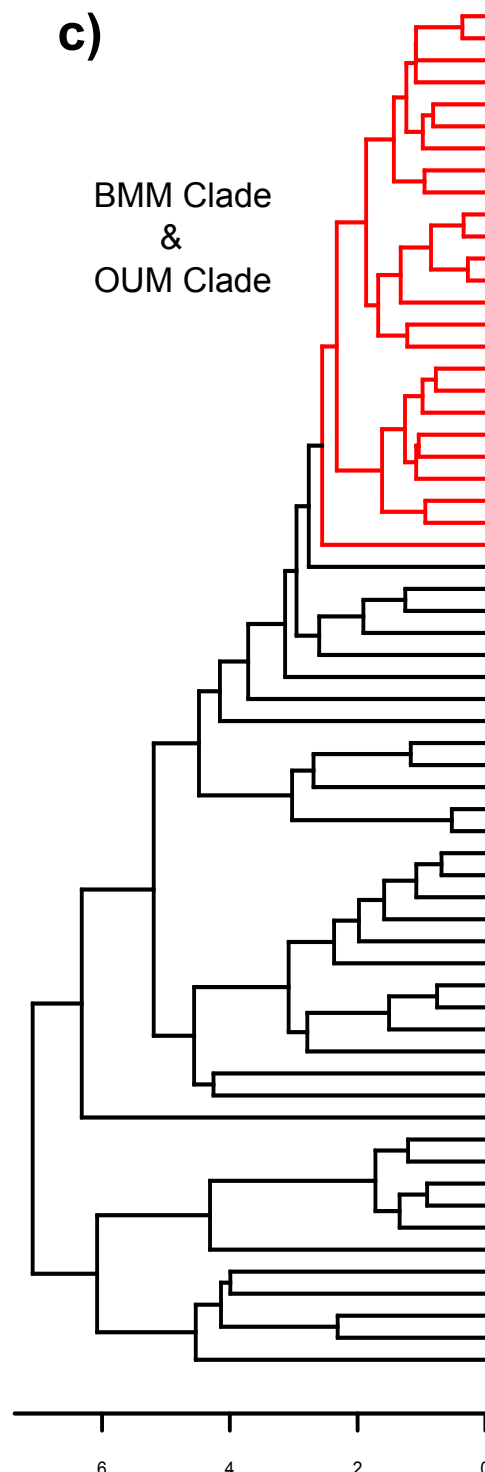**d)**BMM Geo  
&  
OUM Geo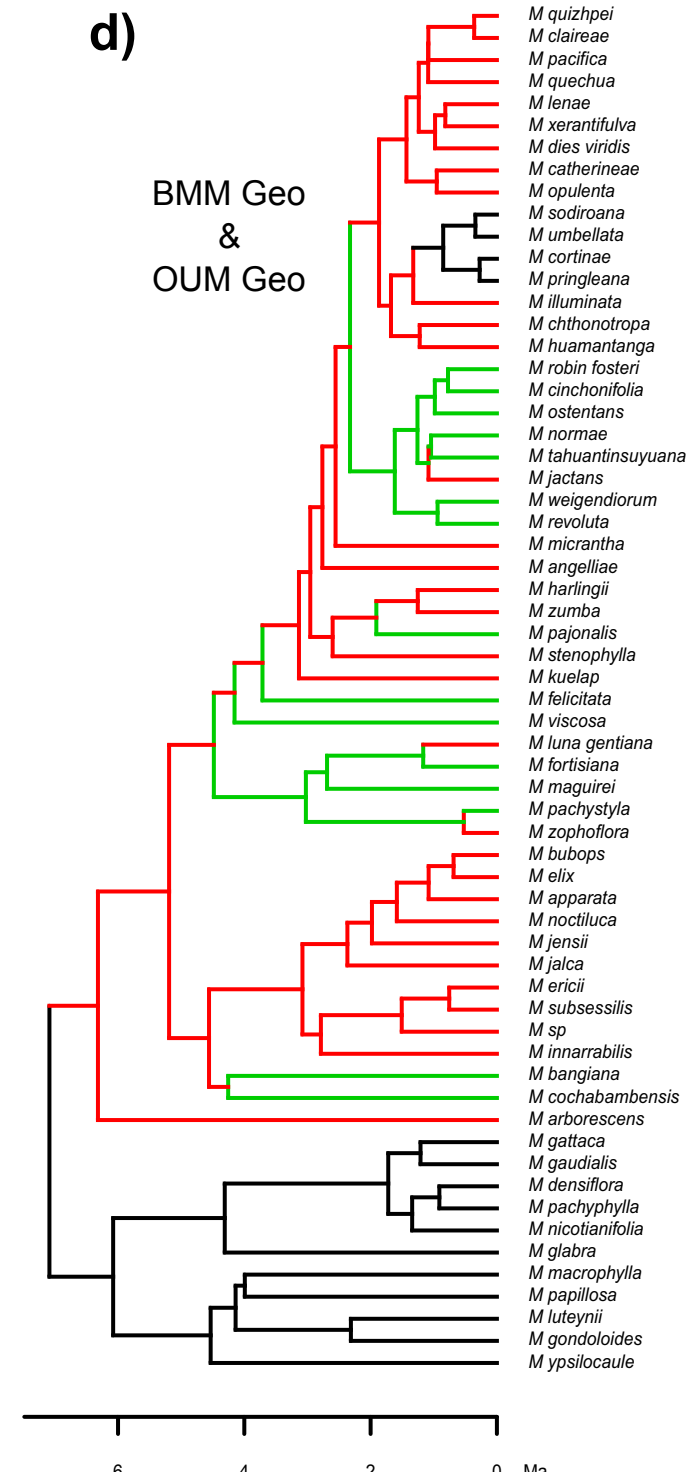

Supplement: Supplementary file 1 [file biology-10-00825-s001.zip › Figure_S1.pdf]

a)

SHIFT  
&  
ER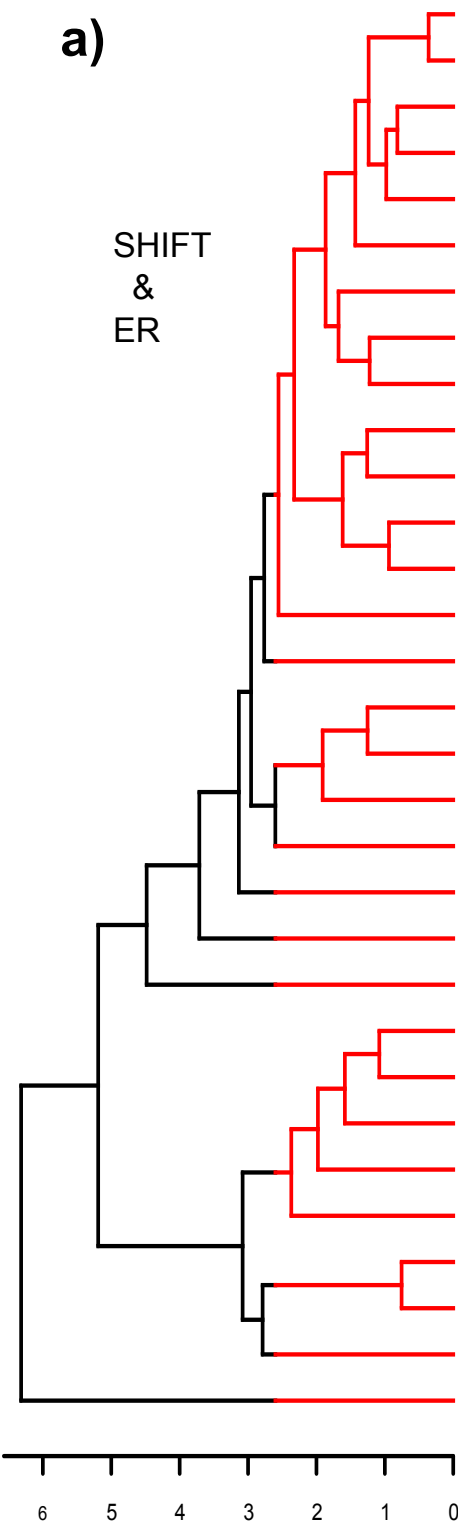

b)

BMM Regime  
&  
OUM Regime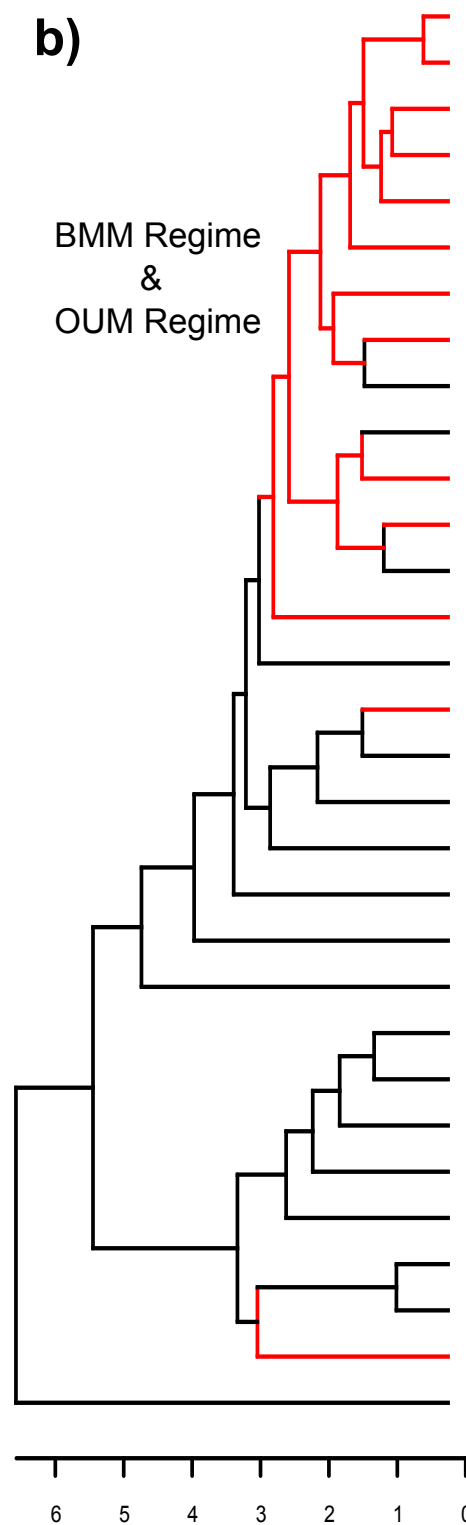

c)

BMM Clade  
&  
OUM Clade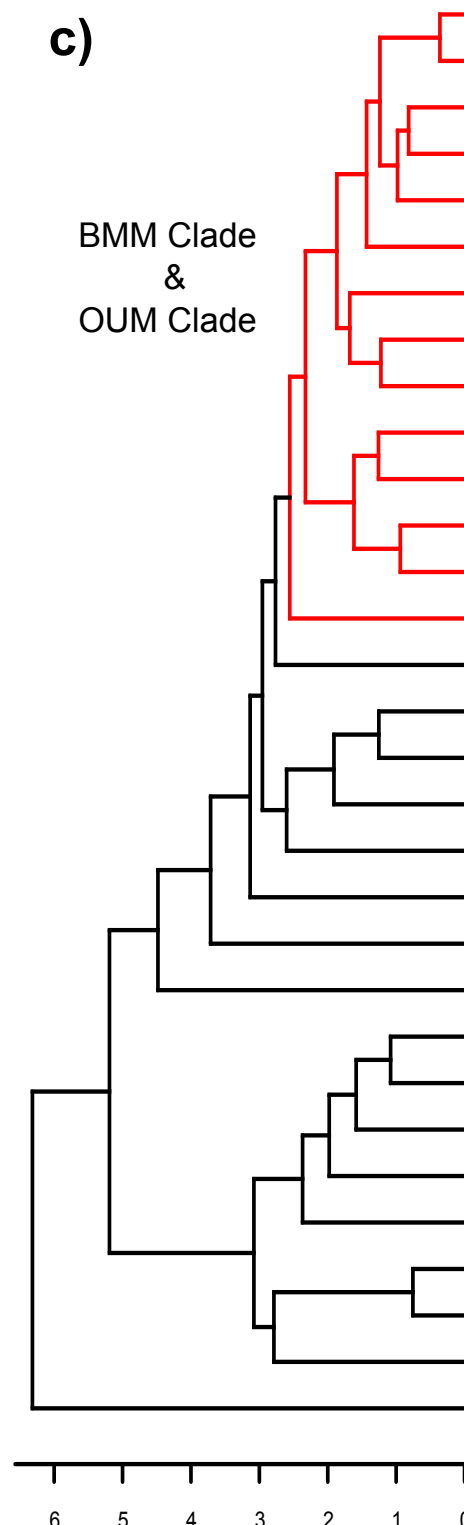

d)

BMM Geo  
&  
OUM Geo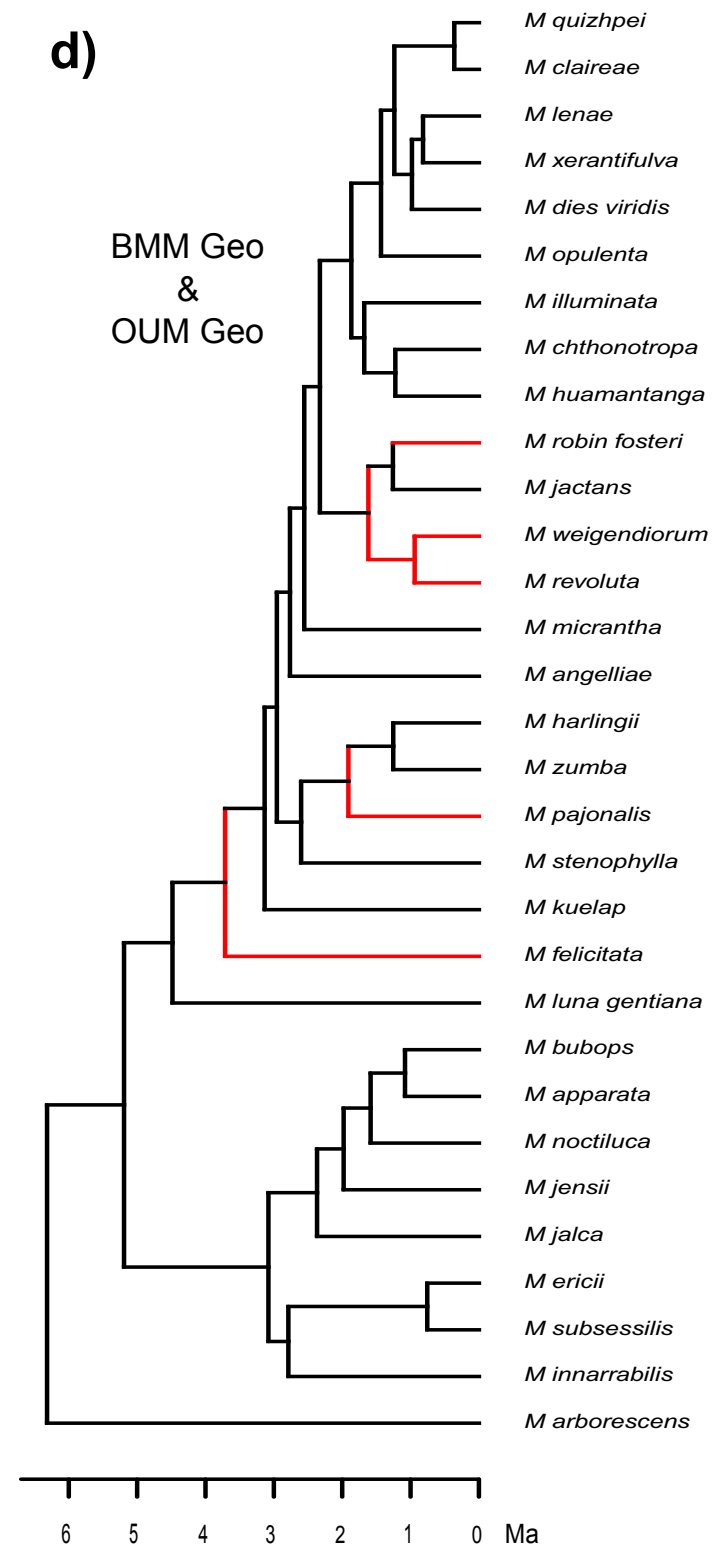

Supplement: Supplementary file 1 [file biology-10-00825-s001.zip › Figure_S2.pdf]

**Leaf size**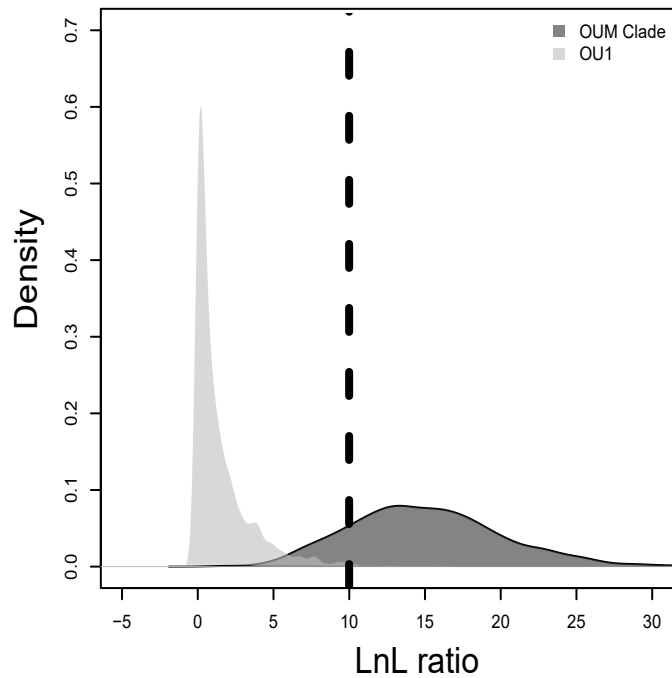**Flower size**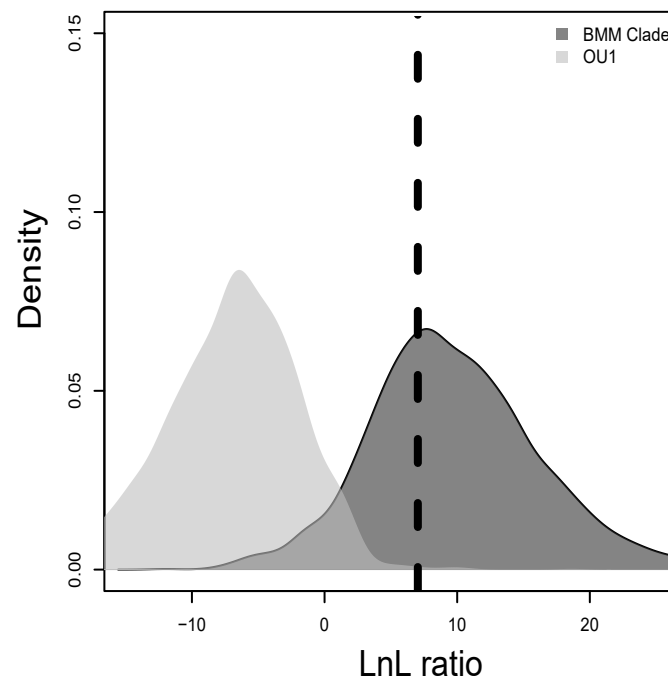**Plant size**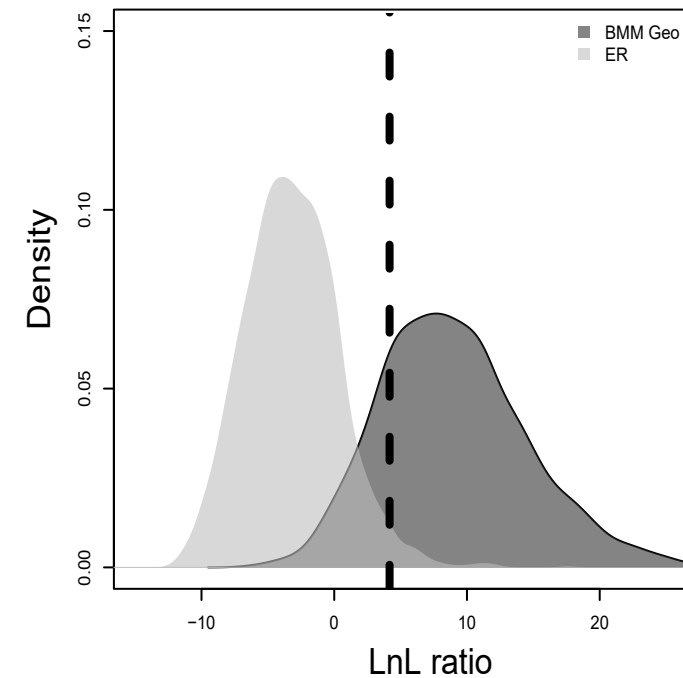**Specific Leaf Area**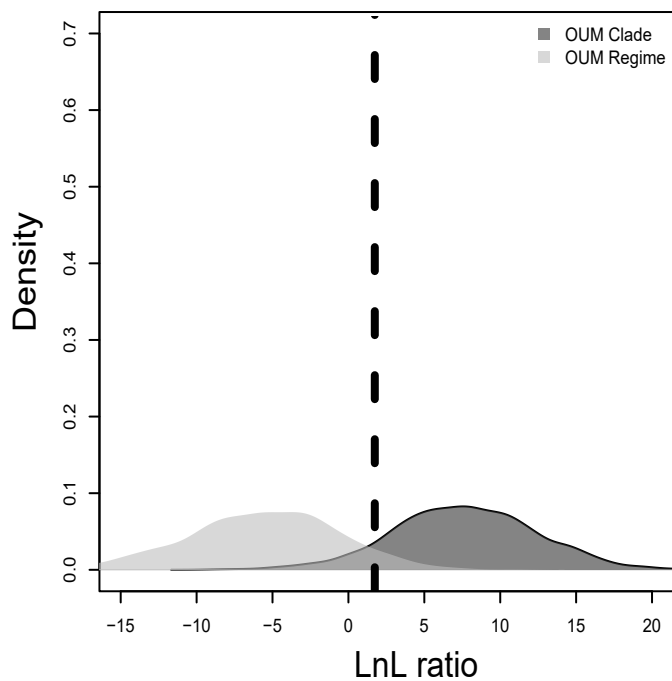**Altitudinal niche**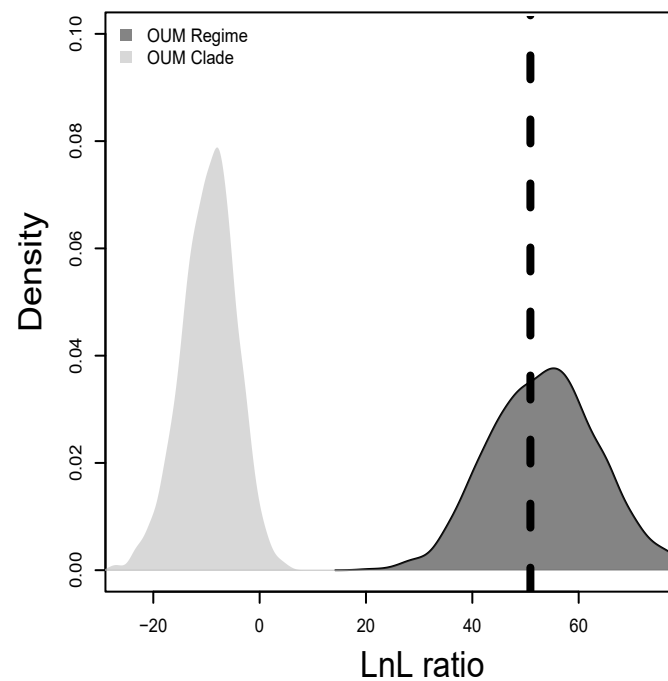**Latitudinal niche**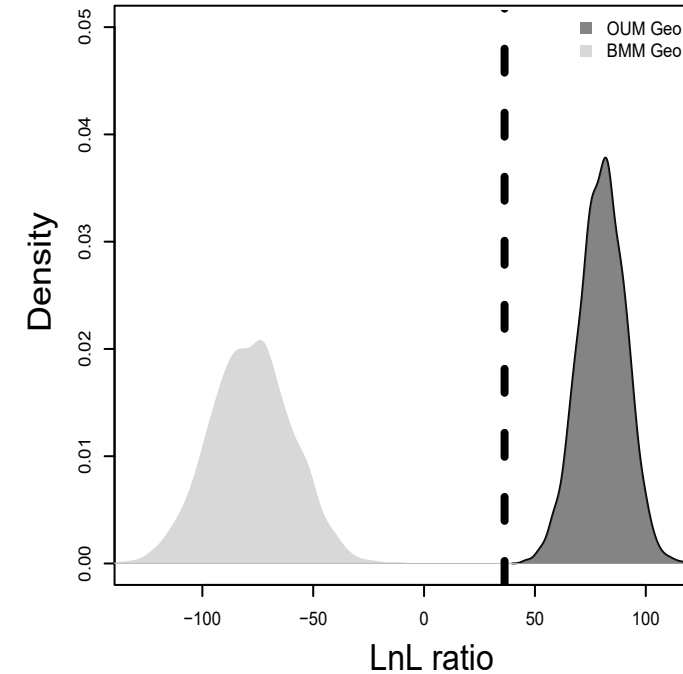

Supplement: Supplementary file 1 [file biology-10-00825-s001.zip › Figure_S3.pdf]
